# A new-engineered integrative tool to target the terminal compartment of the *Streptomyces* chromosome

**DOI:** 10.1007/s00253-026-13707-2

**Published:** 2026-02-21

**Authors:** Nicolas Delhaye, François R. Pélé, Hoda Jaffal, Sylvie Lautru, Hervé Leh, Stéphanie G. Bury-Moné

**Affiliations:** Institute for Integrative Biology of the Cell (I2BC), Université Paris-Saclay, CEA, CNRS, 91198 Gif-Sur-Yvette, France

**Keywords:** Serine-integrase, *Streptomyces*, Genetic tools, Spatial compartments, Phage, Genetic engineering

## Abstract

**Abstract:**

Phages are a valuable resource for the genetic engineering of *Streptomyces* antibiotic-producing bacteria. Indeed, a few integrative vectors based on phage integrase are available to insert transgenes at specific genomic loci. Chromosome conformation captures previously demonstrated that the *Streptomyces* linear chromosome is organized in two spatial compartments: the central compartment encompassing the most conserved and highly expressed genes in exponential phase, and the terminal compartments enriched in poorly conserved sequences including specialized metabolite biosynthetic gene clusters. This study introduces a new integrative tool based on a recently described phage, Samy, which specifically targets the terminal compartment of its native host chromosome. Samy is related to PhiC31 phage and, like the latter, encodes a serine integrase. Whereas PhiC31 targets a site generally located near the origin of replication, the Samy integration site is one of the farthest known *attB* sites from it. We demonstrated that the Samy integrase efficiently mediates the specific integration of a non-replicating plasmid in six *Streptomyces* strains from distinct clades. Bioinformatic analyses revealed that the Samy-*att*B site is rather conserved and located in the terminal compartment of most *Streptomyces* chromosomes. Finally, heterologous expression of the albonoursin biosynthetic gene cluster from the Samy-, PhiC31-, and R4-*attB* sites yields quantitatively equivalent levels of production, though qualitative differences were observed. Altogether, these results demonstrate that the *att-int* Samy system expands *Streptomyces* genetic engineering tools by enabling targeted integration in the terminal chromosomal compartment.

**Key points:**

• *Samy-based integrative vectors are new tools for engineering Streptomyces strains.*

• *They target the terminal compartment, farthest from the origin in most strains.*

• *They facilitate efficient heterologous production of the albonoursin antibiotic.*

**Supplementary Information:**

The online version contains supplementary material available at 10.1007/s00253-026-13707-2.

## Introduction

*Streptomyces* are renowned for their prolific production of specialized metabolites, including antibiotics, pesticides, or pigments, with applications in medicine, agriculture, and the food industry. These bacteria are also well-known for their biodegradation efficiency, driven notably by the secretion of potent enzymes. Therefore, their genome engineering is therefore of great biotechnological interest (Lee et al. 2019; Mitousis et al. 2020). Temperate phages and integrative plasmids serve as crucial tools for the site-specific, stable, and possibly multiplexed insertions (Li et al. 2017, 2019; Gao and Smith 2021) of (over)expression cassettes into genomes. This capability is essential for the long-term, large-scale biotechnological exploitation of *Streptomyces* strains. Orthogonal integration systems for *Streptomyces* genomes have been developed, based on tyrosine-recombinases [pSAM2 (Boccard et al. 1989; Raynal et al. 1998), VWB (Van Mellaert et al. 1998), µ1/6 (Farkašovská and Godány 2012)] or serine-recombinases [PhiC31 (Bierman et al. 1992; Groth et al. 2000), PhiBT1 (Gregory et al. 2003), TG1 (Morita et al. 2009), R4 (Miura et al. 2011), SV1 (Fayed et al. 2014), PhiJoe (Fogg et al. 2017), PhiOZJ (Ko et al. 2020), PhiWTR (Ko et al. 2020)]. These enzymes, also named “integrases,” catalyze the specific recombination between *att*P (attachment site on the phage or plasmid) and *att*B (attachment site on the bacteria) sequences, generating *att*L (attachment site on the left) and *att*R (attachment site on the right) sites on either side of the integrated construct [for reviews, see (Merrick et al. 2018; Kormanec et al. 2019; Mitousis et al. 2020; Ba et al. 2023; Smith 2015)]. Site-specific recombination systems based on serine-integrases are considered the simplest and more likely to function across a broader range of cells due to the absence of host factor requirement (Merrick et al. 2018; Ba et al. 2023). The integration is generally considered directional, meaning that the reverse reaction (*att*L x *att*R recombination) occurs only in the presence of specific partners known as recombination directionality factors (RDFs). However, some evidence challenges this assumption, with characterization of the PhiJoe and PhiBT1 integrases (Zhang et al. 2008; Fogg et al. 2017) and sensitive excision assays revealing activity in PhiC31 as well (Duan et al. 2025).

The *Streptomyces* chromosome is linear—which is unusual for bacteria—and genetically compartmentalized into a central region harboring the origin of replication and core genes and two extremities populated by poorly conserved sequences (Bury-Moné et al. 2023). These extremities are enriched in genomic islands including specialized metabolite biosynthetic gene clusters (SMBGCs). The majority of these poorly conserved sequences remain transcriptionally silent and/or weakly expressed under lab conditions (Van Bergeijk et al. 2020; Lioy et al. 2021). As reported in *Streptomyces ambofaciens* ATCC 23877 (Lioy et al. 2021) and *Streptomyces coelicolor* A3(2) (Deng et al. 2023), the genetic compartmentalization correlates with chromosome architecture and gene expression: The distal ribosomal RNA (*rrn*) operons delimit a highly structured and expressed region termed “central compartment,” presenting structural features distinct from those of the terminal compartments which are almost transcriptionally quiescent during vegetative growth. This architecture and gene expression pattern are dynamic during the *Streptomyces* developmental cycle (Lioy et al. 2021; Szafran et al. 2021; Deng et al. 2023).

The central compartment is considered as a hotspot for integrative element insertions (Choufa et al. 2022). In line with this observation, a systematic analysis of the location of remnant and complete prophage sequences along *Streptomyces* chromosome revealed that they are most commonly found in the half that includes the origin of replication (Sharma et al. 2023). Accordingly, the *Streptomyces* integrative vectors generated so far target the central compartment in most strains. These include site-specific recombination systems based on pSAM2 (Boccard et al. 1989; Raynal et al. 1998), PhiC31 (Bierman et al. 1992; Groth et al. 2000), VWB (Van Mellaert et al. 1998), PhiBT1 (Gregory et al. 2003), TG1 (Morita et al. 2009), µ1/6 (Farkašovská and Godány 2012), SV1 (Fayed et al. 2014), PhiJoe (Fogg et al. 2017), PhiOZJ (Ko et al. 2020), and PhiWTR (Ko et al. 2020), with the three last targeting the same *att*B site. The notable exception to this is the R4-integrase-based plasmids (Miura et al. 2011), which target a site located in the terminal spatial compartment of *Streptomyces* chromosome in most strains (Lorenzi et al. 2022).

We recently identified a new temperate phage named Samy, which is closely related to PhiC31 (Jaffal et al. 2024). Samy specifically targets the terminal compartment of the native host chromosome in *S. ambofaciens* ATCC 23877. While its prophage form is mainly silent under most growing conditions analyzed so far, the identification of its induction conditions revealed that the Samy productive cycle promotes the in vitro dispersal of *Streptomyces* multicellular structures in response to stress (Jaffal et al. 2024). In this study, we characterized a site-specific recombination system based on Samy serine-integrase. This expands the genetic toolkit for engineering the *Streptomyces* chromosome by broadening the range of targeted locations. To date, this new tool is capable of targeting the most distant site within the terminal compartment of most *Streptomyces* strains.

## Materials and methods

### Strains, plasmids, and growth conditions

The strains and plasmids used in this study are listed in Table S1, while all primers are provided in Table S2. *Streptomyces* strains were grown at 30 °C on solid soy flour-mannitol (SFM) medium (20 g/L organic soy flour, 20 g/L mannitol, 20 g/L agar) (Kieser et al. 2000), in liquid Tryptone Soya Broth (TSB, 30 g/L Tryptic Soy Broth BD™ 211,825) (Kieser et al. 2000), or in liquid MP5 (7 g/L yeast extract, 20.9 g/L MOPS, 5 g/L NaCl, 1 g/L NaNO_3_, 36 mL/L glycerol; pH 7.5) (Pernodet et al. 1993). The 2TY (16 g/L Bacto Tryptone, 10 g/L Yeast Extract, 5 g/L NaCl) and SNA (8 g/L Bacto Nutrient Broth) media (Kieser et al. 2000) were used exclusively for conjugation. When appropriate, apramycin (50 µg/mL) and/or nalidixic acid (50 µg/mL) were added to the growth media. *Escherichia coli* strains were grown at 37 °C in Luria-Bertani (LB) medium, supplemented with 50 μg/mL apramycin and/or 25 µg/mL kanamycin when appropriate.

For analyses of growth rate and albonoursin production, approximately 5.10^6^ spores were inoculated into 50 mL of MP5 liquid medium in a 500 mL baffled Duran® Erlenmeyer flask with a silicon stopper. Cultures were incubated at 30 °C with orbital shaking at 180 rpm (INFORS Unitron standard) and harvested during the exponential (≈ 24 h–30 h) or stationary (48 h, 72 h, and 96 h) phases. In this medium, bacterial growth was monitored using pseudo-opacimetry (Biochrom WPA CO7500 colorimeter, Fisher Scientific). The term “pseudo-opacimetry” reflects the limitations of this method due to the multicellular nature of these bacteria, which can affect accuracy. However, in MP5 medium, bacterial growth is sufficiently dispersed to allow reliable monitoring with this approach. The generation time was calculated from the exponential growth phase data using the equation *N*_t_ = *N*_0_.*e*^μt^, where *μ* represents the growth rate. To account for minor variations in latency phase duration between spore stocks, growth curves were normalized by aligning the end of the latency phase to 24 h, corresponding to a pseudo-opacimetry of 0.03 (which equates to an OD₆₀₀ of ~1, typically reached at 33 h with a mean generation time of 1.8 h for *S. lividans* strains).

To assess the stability of apramycin resistance in strains harboring pALB vectors (*att-int* PhiC31, R4, or Samy), spore stocks were initially prepared on SFM media without antibiotics. For each genetic background, three independent clones underwent three successive weekly passages on antibiotic-free SFM plates. At each passage, spores were collected, and the stocks were titrated by comparing colony-forming units (CFU) on SFM medium with and without apramycin.

### Vector cloning

Standard techniques were used for recombinant DNA manipulation. BsaI sites were introduced into pOJ260 by PCR amplification using SBM375 and SBM376 primers, generating pOJ260-MoClo. In parallel, a PCR product corresponding to the 56,510–58,494 genomic region of Samy phage (OR263580.1), including the *SAMYPH94* gene encoding Samy-integrase, its promoter, and the Samy-*att*P site sequences flanked by BsaI sites, was obtained by amplifying the Samy genome with SBM560 and SBM561 primers. The Samy genome was isolated from the supernatant of *S. ambofaciens* ATCC 23877 grown in BM (bacteriophage medium), as previously described (Jaffal et al. 2024). This insert was then cloned into pOJ260-MoClo via Golden Gate Assembly (NEBridge® Golden Gate Assembly Kit BsaI-HF® v2) according to the manufacturer’s recommendations.

A pOSV vector from the Aubry et al*.* collection (Aubry et al. 2019) conferring apramycin resistance was digested with AflII and SbfI. In parallel, a PCR product corresponding to the 56,510–58,494 genomic region of Samy phage (OR263580.1), flanked by AflII and SbfI sites, was obtained by amplifying the Samy genome with SBM588 and SBM589 primers. After purification and digestion with AflII and SbfI, both the vector and insert were heat-inactivated and ligated using T4 DNA ligase (PROMEGA). Similarly, the R4 *att-int* module from pJH1R4 (Gao et al. 2019) was amplified by PCR using the HL-R4-AflII and HL-R4-SbfI primers and cloned between the AflII and SbfI sites, replacing the PhiC31 *att-int* system in the pOSV802 vector and yielding the pOSV819 plasmid.

We previously constructed the plasmid pCEA007, which harbors the albonoursin BGC (*albA*, *albB*, and *albC*) under the control of the *rpsL*(TP) promoter and the *tipA* ribosome binding site (Aubry et al. 2019). We replaced the PhiC31 *att-int* system in pCEA007 with the R4 *att-int* module from pOSV819 and the Samy *att-int* module from pOSV876 by cloning them into the SpeI-SbfI sites. For clarity, the integrative vectors designed for chromosomal integration of the albonoursin biosynthetic gene cluster at the PhiC31-, R4-, and Samy-*att*B sites were designated “pALB-PhiC31” [synonymous with pCEA007 (Aubry et al. 2019)], “pALB-R4,” and “pALB-Samy,” respectively.

### Conjugation

Plasmids intended for conjugation into *Streptomyces* were first introduced by electroporation into *E. coli* ET12567 carrying pUZ8002 to provide the necessary transfer functions. Conjugation was then performed as previously described (Kieser et al. 2000). Briefly, 10⁸ *Streptomyces* spores were inoculated in 0.5 mL of 2TY medium, incubated at 50 °C for 10 min to induce germination, and then maintained at 30 °C for 2–3 h until the donor cells were ready. *E. coli* donor cells were grown in LB medium with kanamycin and apramycin until reaching an OD₆₀₀ of 0.4–0.6, then washed three times with cold LB. Five hundred microliters of *Streptomyces* recipient cells were mixed with an equal volume of donor cells and centrifuged to form a pellet. The cell pellet was transferred to a 22-mL SFM plate containing 10 mM MgCl₂. After overnight incubation at 30 °C—except for *Streptomyces venezuelae*, which was incubated at room temperature—4.5 mL of SNA medium with nalidixic acid and apramycin was added to the plates, thereafter incubated at 30 °C for 5–7 days. For subsequent analysis, at least three independent exconjugant clones were reisolated on SFM plates supplemented with nalidixic acid and apramycin, then expanded to generate spore stocks. Genomic DNA was extracted from the strains using a phenol–chloroform method and subjected to PCR analyses to verify proper genomic organization (Table S2).

### Experimental identification of Samy-*att*B sites in six strains of interest

For each species, three apramycin-resistant exconjugants derived from the conjugation of pOJ260-*SAMYPH94* were isolated and characterized at the genomic level as follows. First, for five strains (*S. ambofaciens* DSM 40697, *Streptomyces albidoflavus* J1074, *Streptomyces globisporus* NBC_01004, *Streptomyces lividans* TK24, and *S. venezuelae* ATCC 10712), one clone per strain was subjected to high-throughput sequencing (Plasmidsaurus) to confirm the unique insertion of the vector (Data S1). Next, the insertion identity in three independent clones per strain—covering the five species listed above plus *S. coelicolor* A3(2)—was verified by PCR on genomic DNA (gDNA). The *att*L site was amplified using an SBM585 primer and a species-specific primer, while the *att*R site was amplified using an SBM584 primer and a species-specific primer (Table S2). PCR products were analyzed on a 1% agarose gel with ClearSight DNA Stain (Bioatlas). The Thermo Scientific GeneRuler 1 kb Plus DNA Ladder was used as the molecular size marker.

### Analysis of Samy-based vector excision

Three independent clones carrying an integrated form of pOJ260-*SAMYPH94* and three independent stocks of the *S. ambofaciens* ATCC 23877 strain were grown for three days in TSB medium before being collected for phenol extraction of gDNA. To prevent amplification bias due to DNA topology (Hou et al. 2010), both gDNAs and the pOJ260_*SAMYPH94* plasmid were linearized using the SalI restriction enzyme (NEB). Amplification was performed on 5 ng of digested gDNA in a final volume of 10 µL using the LightCycler® 480 SYBR Green I Master (Roche Diagnostics). The primers used to amplify Samy-*att*P junction and *SAMYPH94* gene are listed in Table S2. qPCR efficiencies and the absolute number of amplified copies were determined using a standard curve ranging from 3.10^2^ to 3.10^6^ copies of pOJ260_*SAMYPH94* plasmid. The proportion of the plasmid in its excised form was calculated by dividing the *att*P junction copy number by the *SAMYPH94* gene copy number.

### Promoter prediction

We used PromPredict (Rangannan and Bansal 2009) (PromPredict_genome_V1.exe) and G4PromFinder-v.2.1 (Di Salvo et al. 2018) to predict promoters within the sequence cloned into pOJ260-*SAMYPH94* and pOSV876 vectors, as well as in the region located upstream *SAMYPH94* following its integration at the *att*B site in the *S. ambofaciens* ATCC 23877 genome (GCF_001267885.1). PromPredict_genome_V1.exe was run with default parameters and a window of 50 bp. Please note that *SAMYPH94* (OR263580.1: 56925–58457) is also referred to as *SAM23877_RS39280* in its prophage form within the genome of *S. ambofaciens* ATCC 23877 (NZ_CP012382.1: 6589832–6591364).

### Bioinformatic analysis of Samy target gene

**Fig. 1:**
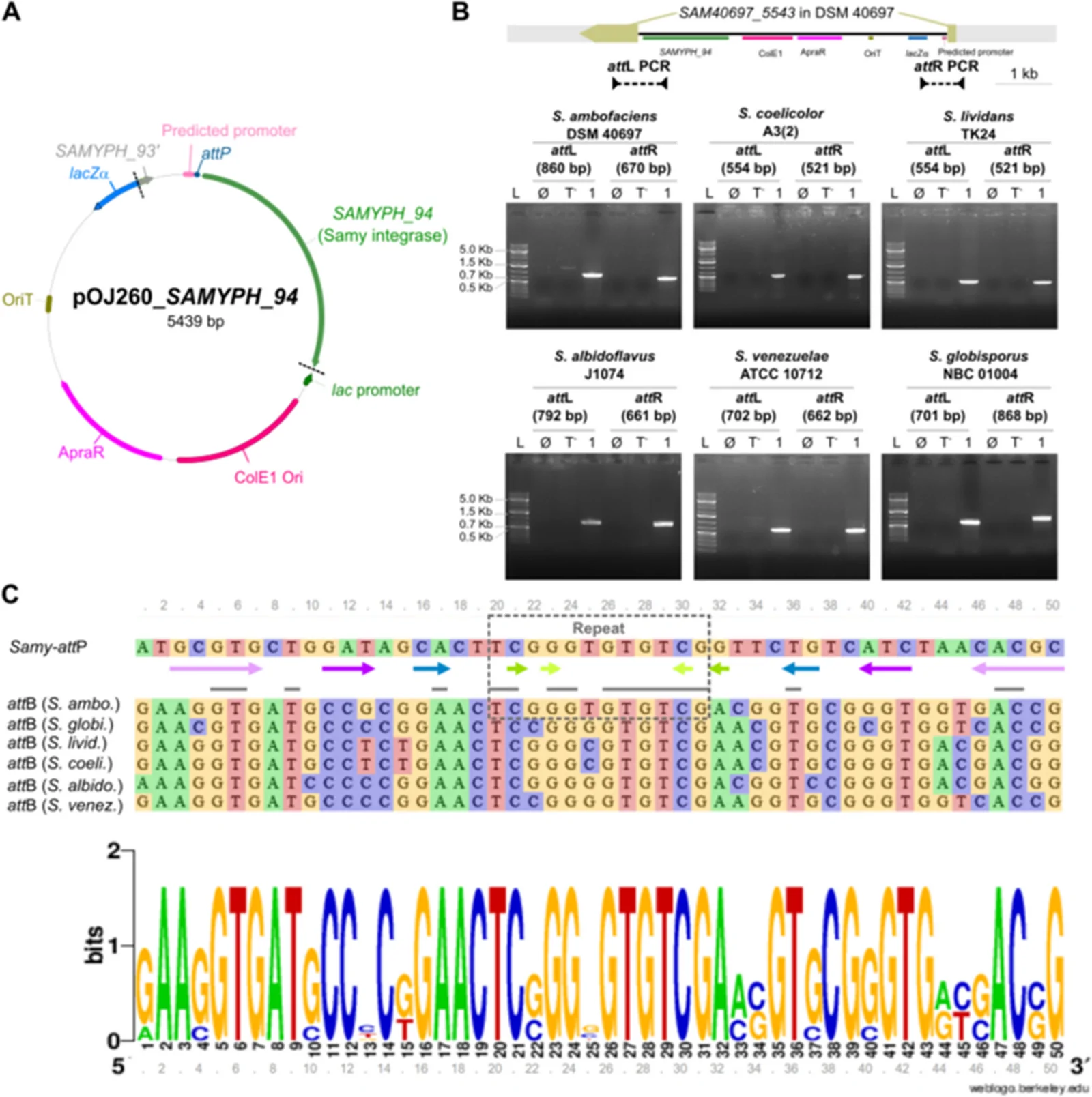
Characterization of Samy phage attachment and integration sites in a panel of six *Streptomyces* species. **A** Schematic representation of pOJ260_*SAMYPH94* vector. The two black dashed lines mark the boundaries of the Samy phage region, which was cloned into the pOJ260 vector (Bierman et al. 1992) using a Golden Gate Assembly approach. The predicted promoter and *att*P site sequences are presented in Figure S1. ApraR, apramycin resistance; ColE1 Ori, ColE1 origin of replication in *E. coli*; *lacZα*, gene encoding the LacZ alpha peptide; OriT, origin of transfer; *SAMYPH93’*, fragment of *SAMYPH93* gene). **B** Integration of pOJ260_*SAMYPH94* into the *SAM40697_5543* gene or its orthologs in a panel of six *Streptomyces* species. PCR amplification of the *att*L and *att*R regions defined based on Samy prophage orientation in its native *S. ambofaciens* ATCC 23877 host is shown, with expected sizes indicated in brackets. The integration of pOJ260_*SAMPHY_94* in *S. ambofaciens* DSM 40697 is represented at scale. No PCR amplification is expected when gDNA from wild-type *Streptomyces* strains is used as the template (“T-”) or in absence of template (“∅”). The number “1” represents clone #1 analyzed for each strain. “L” denotes the molecular weight ladder (Thermo Scientific GeneRuler DNA Ladder 1 Kb Plus). The results for three independent clones per strain are presented in Figure S2. **C** Alignment of Samy-*att*P and *att*B sites from six *Streptomyces* strains against the consensus *att*B sequence. Sequences of 50 bp centered on the repeat (black hatched rectangle) identified in the integrated form of the Samy prophage (Jaffal et al. 2024) were analyzed. Strain names are presented in abbreviated form before each sequence, with the full names provided in panel B. The logo was generated using the online tool WebLogo (https://weblogo.berkeley.edu/logo.cgi) (Crooks et al. 2004). Gray lines indicate positions that are perfectly conserved across all analyzed sequences. The *att*P site is surrounded by imperfect inverted repeat sequences shown as arrows of the same color, indicating complementary sequences. During integration, a minimal sequence of 2-bp identical between *att*P and *att*B sites is referred to as the crossover sequence (Smith 2015). The repeat region contains several identical 2 bp positions, leaving the exact cleavage site yet to be determined. Please note that *S. coelicolor* and *S. lividans att*B sites are identical

**Fig. 2:**
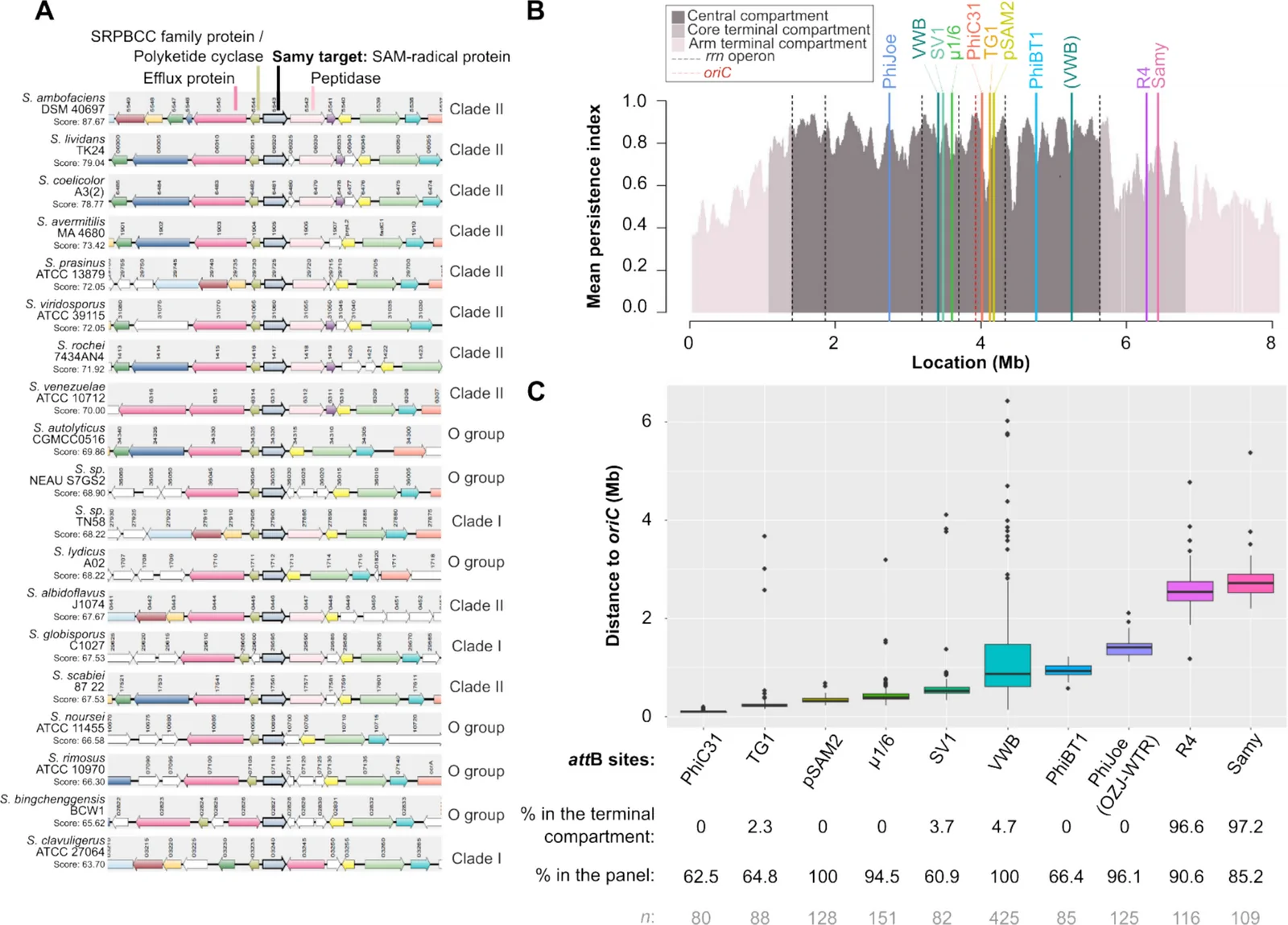
Comparative genomics for the analysis of Samy-*att*B sites in a panel of *Streptomyces*. **A** Analysis of gene synteny around the Samy-*att*B site in a panel of 20 *Streptomyces* strains from Clade I, Clade II, and the “O’ group. The genes encoding the SAM40697_5543 query protein and its orthologs are drawn in bold. The color code allows the identification of orthologs and paralogs in the different strains. Build using SyntTax (https://archaea.i2bc.paris-saclay.fr/synttax/) (Oberto 2013). *SAM40697_5545* and *SAM40697_5544*, located upstream of *SAM40697_5543*, encode a major facilitator superfamily (MFS) transporter related to multidrug resistance and a polyketide cyclase from the SRPBCC family, respectively. Downstream, *SAM40697_5542* encodes a putative secreted peptidase. **B** Location along *S. ambofaciens* DSM 40697 chromosome of the *attB* sites for all *att-int* systems described to date for engineering the *Streptomyces* chromosome. The level of gene persistence along the chromosome of *S. ambofaciens* DSM 40697 obtained from (Lorenzi et al. 2022) is represented using a sliding window (81 coding sequences (CDSs), with 1 CDS steps). The positions of all *rrn* operons and of the origin of replication are indicated by dashed black and red lines, respectively. The central compartment (dark gray) is delimited by the distal *rrn*. Terminal compartments are highlighted in dark pink when they include core genome genes, and in light pink when they do not. Sites that deviate from the consensus sequence (coverage < 80%) are shown in brackets. Figure S4 presents the same data for four additional strains of interest. **C** Distance of *att*B sites from the origin of replication across 128 *Streptomyces* strains. The positions of the sites in a panel of 128 strains were determined using BlastN, with the Samy-*att*B sequences identified in this study and all characterized sites from *Streptomyces att-int* systems (Table S3). Using the OriFinder software to identify the origin of replication, the distance from the origin to each of these sites was calculated. All the boxplots represent the first quartile, median and third quartile. The upper whisker extends from the hinge to the largest value no further than 1.5 * the inter-quartile range (IQR, i.e., distance between the first and third quartiles). The lower whisker extends from the hinge to the smallest value at most 1.5 * IQR of the hinge. Below each boxplot, the number of sites included in the analysis (*n*) is displayed, along with the percentage of sites located in the terminal compartment (“% in the terminal compartment”) and the percentage of strains in the panel harboring at least one given site (“% in the panel”)

The Samy-*att*B logo was generated using the online tool WebLogo (https://weblogo.berkeley.edu/logo.cgi) (Crooks et al. 2004). The gene persistence index was retrieved from Lorenzi et al.’s publication (Lorenzi et al. 2022). Gene synteny between bacterial genomes was analyzed using SyntTax (https://archaea.i2bc.paris-saclay.fr/synttax/) (Oberto 2013) against 19 strains listed in Fig. 2A. The percentages of identity and similarity between SAM40697_5543 from *S. ambofaciens* DSM 40697 (WP_063483567.1) and SplB from *B. subtilis* strain 168 (A0A6M3ZAI9) were determined by the Stretcher program, available online (https://www.ebi.ac.uk/jdispatcher/psa/emboss_stretcher) with default parameters. The protein structures were predicted using AlphaFold3 (Abramson et al. 2024). PyMOL (v.2.6.0a0) was used to align the structures and determine the root-mean-square deviation of atomic positions (RMSD). The prediction of *att*B sites was carried out in a panel of 128 genomes corresponding to a panel of 126 genomes representative of the diversity of the genus previously described (Lorenzi et al. 2022), plus 2 strains of interest (*S. lividans* TK24, *S. globisporus* NBC_01004). We used BlastN (ncbi-blast-2.14.0+) to identify sequences similar to the 10 *att*B sites listed in Table S3, as well as the four Samy-*att*B sites experimentally identified in *S. albidoflavus* J1074, *S. globisporus* NBC_01004, *S. lividans* TK24, and *S. venezuelae* ATCC 10712 (Fig. 1C). For further analyses, only results with an *e*-value < 8.10^–4^ and a coverage of at least 70% were taken into consideration. The origin of replication was identified using Ori-Finder 2022 (Dong et al. 2022) and used to calculate the distance between each putative *att*B site and *ori*C. For each genome, the central compartment is defined as the region between the distal ribosomal operons, while the terminal compartments correspond to the genome’s extremities beyond these operons, as previously described (Lioy et al. 2021; Lorenzi et al. 2022). Data were analyzed with R software (R Core Team 2023) to generate most graphs and perform statistical tests.

### HPLC analysis of albonoursin cluster products

Around five million spores were inoculated into 50 mL of MP5 liquid medium in a 500-mL baffled Duran® Erlenmeyer flask with a silicon stopper. Cultures were incubated at 30 °C with orbital shaking at 180 rpm (INFORS Unitron standard) for 4 days. After centrifugation, supernatants were filtered using Whatman PVDF Mini-UniPrep syringeless filters (0.2 µm) and analyzed on an Atlantis C18 T3 column (250 × 4.6 mm, 5 µm, 31 °C) using an Ultimate 3000 series HPLC system. Elution was performed with a linear gradient of 0% to 45% solvent B (solvent A, 0.1% HCOOH in H₂O; solvent B, 0.1% HCOOH in CH₃CN) over 45 min at a flow rate of 1 mL/min, and albonoursin BGC products were detected by absorbance at 297 nm. To identify peaks specific to *S. lividans* strains harboring the albonoursin cluster, supernatants from *Streptomyces noursei* ATCC 11455 and *S. lividans* TK24 were used as positive and negative controls, respectively, with uninoculated MP5 medium as an additional negative control. Experiments were conducted across at least three independent trials, each utilizing three independent clones for each *S. lividans* strain carrying the albonoursin biosynthetic gene cluster integrated at either the *attB*-PhiC31 (pALBPhiC31), *attB*-R4 (pALBR4), or *attB*-Samy (pALB-Samy) site.

### Solid-phase extraction (SPE) enrichment of samples

Fifteen milliliters of 0.45 µm-filtered culture supernatant were loaded onto a pre-equilibrated Strata-X 33 µm polymeric reversed-phase C18 SPE column. The columns were washed with 30 mL of a solvent mixture (H₂O, 0.1% formic acid/ACN, 0.1% formic acid; 75:25, v/v). Elution was performed using 300 µL of a solvent mixture (H₂O, 0.1% formic acid/ACN, 0.1% formic acid; 50:50, v/v). The eluted samples were then subjected to mass spectrometry analysis.

### Liquid chromatography-mass spectrometry (LC-MS) experiments

LC-MS analyses were performed using a Bruker Daltonics Esquire HCT ion trap mass spectrometer equipped with an orthogonal atmospheric pressure electrospray ionization (AP-ESI) source. The LC flow was split, directing 10% to the mass spectrometer and 90% to a diode array detector. The ESI source operated in positive mode with a nebulizing gas pressure of 241 kPa, a drying gas flow of 8 L/min, and a drying temperature of 340 °C. Nitrogen served as both the drying and nebulizing gas, while helium was used in the ion trap for ion cooling and fragmentation. Mass spectrometry parameters—including capillary voltage, skimmer voltage, and ion transfer settings—were optimized for detecting compounds within an m/z range of 50–600. For structural characterization, an isolation width of 1 mass unit was applied, and a fragmentation energy ramp was employed to optimize MS/MS conditions. For SPE-enriched samples, elution was carried out using a modified gradient: an initial 2-min isocratic run at 95% buffer A (H₂O, 0.1% formic acid), followed by a linear increase to 25% buffer B (ACN, 0.1% formic acid) over 5 min, and then to 50% buffer B over 20 min.

## Results

### Characterization of the Samy integrase target *att*B sites in a panel of *Streptomyces* species

To evaluate the efficiency and tropism of the Samy-based integration system, we cloned, into the conjugative suicide vector pOJ260 (Bierman et al. 1992), the gene *SAMYPH94* encoding the Samy integrase (510 amino acids) and its upstream intergenic region (Fig. 1A). This latter includes a putative promoter identified using PromPredict software and the “TCGGGTGTGTCG” sequence of the predicted *att*P site (Jaffal et al. 2024) (Fig. S1A). The resulting pOJ260_*SAMYPH94* was tested for chromosomal integration in six *Streptomyces* hosts representative of the genus’s diversity. These included strains from the Clade II (*Streptomyces albidoflavus* J1074, *S. ambofaciens* DSM 40697, *S. coelicolor* A3(2), *Streptomyces lividans* TK24) and the Clade I (*Streptomyces globisporus* NBC_01004, *Streptomyces venezuelae* ATCC 10712) according to MacDonald and Currie classification (McDonald and Currie 2017; Lorenzi et al. 2022) (Table S1). Of note, with an average nucleotide identity of 99.04% (Lorenzi et al. 2022), *S. ambofaciens* DSM 40697 is closely related to the Samy native host, *S. ambofaciens* ATCC 23877, but devoid of this prophage.

After intergeneric conjugation, the gDNA of one clone from five strains (*S. ambofaciens* DSM 40697, *S. albidoflavus* J1074, *S. globisporus* NBC_01004, *S. lividans* TK24, and *S. venezuelae* ATCC 10712) was subjected to high-throughput sequencing to determine the number and location of the pOJ260_*SAMYPH94* insertion sites. In all cases, the plasmid was integrated in a single copy within the gene orthologous to *SAM40697_5543* (Table 1, Data S1), which corresponds to the native location of the complete Samy prophage in *S. ambofaciens* ATCC 23877 (Jaffal et al. 2024). Analysis of the neo-integration site thus allowed to formally determine *att*P, *att*B, *att*L, and *att*R sequences (Table 2). The uniform definition of the left and right attachment sites for all strains was based on the native orientation of the Samy prophage in its host chromosome. The position of the integration site was confirmed by PCR amplification of the predicted *att*L and *att*R regions in three independent conjugants for each strain—covering the five species listed above plus *S. coelicolor* A3(2) (Fig. 1B, Fig. S2)—enabling the definition of an *att*B site logo (Fig. 1C). These results indicate that the Samy-based integrative vector is functional across a diverse range of phylogenetically distant strains, accommodating variations in the *att*B site.

**Table 1:** Location of Samy-integrase target gene in the panel of six *Streptomyces* strains of interest

Strain	Gene name	Chromosomal location*
*S. albidoflavus* J1074	XNR_0446	CP004370.1 [531548–532585 bp]
*S. ambofaciens* DSM 40697	SAM40697_5543	NZ_CP012949 [comp(6432936–6434015 bp)]
*S. coelicolor* A3(2)	SCO6481	AL645882.2 [comp(7170631–7171677 bp)]
*S. globisporus* NBC_01004	OG449_04345	CP109059.1 [999844–1000887]
*S. lividans* TK24	SLIV_06020	NZ_CP009124.1 [1344278–1345324 bp]
*S. venezuelae* ATCC 10712	SVEN_6313	NZ_CP029197 [comp(6889333–6890364 bp)]

^*^The start and end of genes containing the Samy-*att*B site are indicated below the chromosome identifier of each strain. “comp” indicates that the gene is in antisense/complementary orientation

**Table 2:**
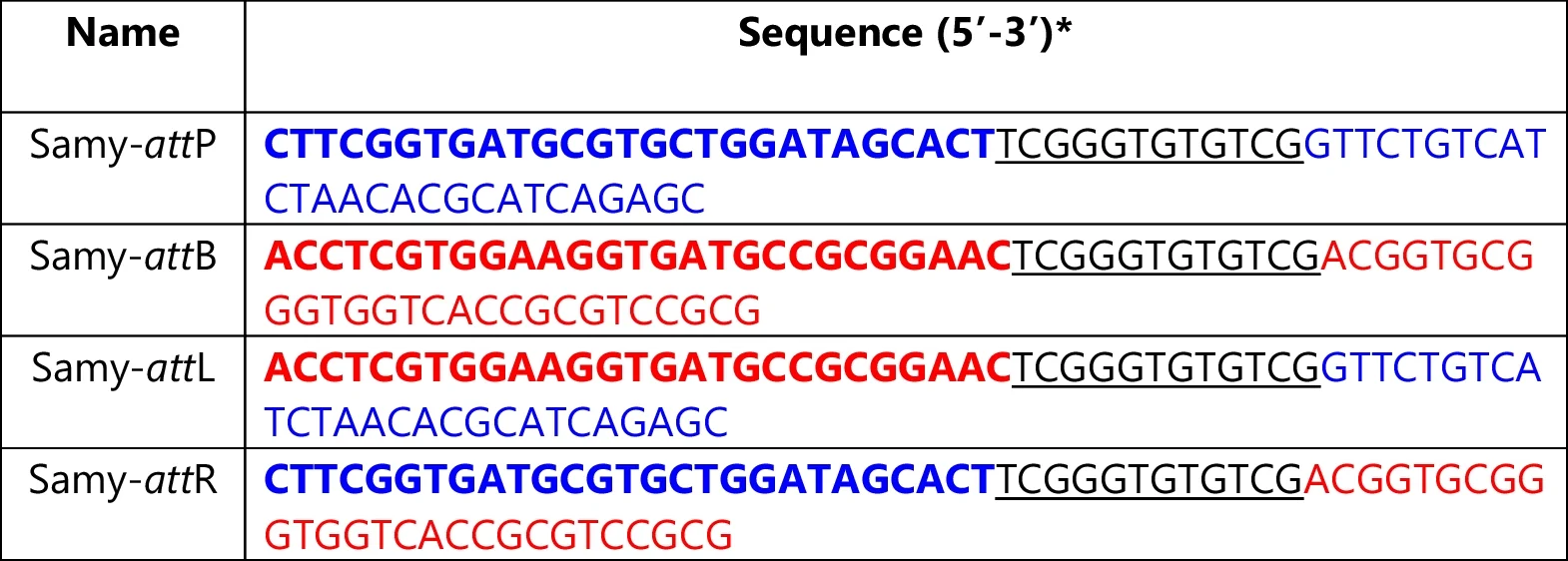
Sequences of the Samy integrase attachment sites in the phage and in the *S. ambofaciens *chromosome

*The repeat sequence observed on either side of the integrated prophage or vector is highlighted. The sequence size was set at 50 bp, consistent with the sizes typically described for serine integrase attachment sites. The writing style varies on either side of the repeat

### Bioinformatic analysis of the Samy-*att*B integration site

The genes targeted by Samy integrase, *SAM40697_5543* and its orthologs, encode a radical S-adenosyl-L-methionine (SAM) protein belonging to the Rv2578c family. This iron-sulfur cluster binding protein is annotated as a putative SplB (spore photoproduct lyase B) in several strains. The SplB protein is involved in DNA repair in *Bacillus subtilis* (Slieman et al. 2000). The similarity with this protein is rather low: SAM40697_5543 and the *B. subtilis* SplB proteins share 17.6% identity and 29.2% similarity over 387 amino acids, with 18.9% gaps. Since proteins with sequence identity below 25% can still have similar structures, we compared the 3D structures of SAM40697_5543 and SplB from *B. subtilis* using AlphaFold3 (Fig. S3). Given that the RMSD between these structures exceeds 4 Å, the annotation of Samy’s target gene as SplB should be interpreted with caution and requires experimental validation.

The persistence of *SAM40697_5543* gene in a previously characterized panel of 127 *Streptomyces* strains is around 88% (Lorenzi et al. 2022). Furthermore, we observed that the synteny around this gene is relatively well conserved in a panel of twenty strains chosen in Clade I, Clade II, and group “O” (others) to represent the *Streptomyces* genus diversity (McDonald and Currie 2017; Lorenzi et al. 2022) (Fig. 2A). *SAM40697_5543* orthologs are frequently co-conserved with their upstream divergent gene, which encodes a putative polyketide cyclase. In certain strains such as *S. ambofaciens* DSM 40697, the target gene of the Samy integrase could possibly be expressed in an operon with genes encoding a peptidase (Fig. 2A). In this case, the integration of Samy might have a polar effect on the expression of the downstream gene.

Finally, we conducted a BlastN analysis to determine the localization of Samy-*att*B in a panel of 128 genomes representative of the genus diversity. Using the *att*B sites identified in our strains of interest as input (Fig. 1C), Samy-*att*B sites were predicted in 85.2% of the strains, as a single copy (Table S4). In parallel, we have also analyzed the positions within these genomes of all the other integration systems described for genetically modifying *Streptomyces* (Table S3). This analysis reveals that the Samy-*att*B site is located in the terminal compartment of most of the analyzed strains (Table S4), with only a few exceptions (*Streptomyces bingchenggensis* BCW 1, *Streptomyces fungicidicus* TXX3120, *Streptomyces sp.* 11 1 2) whose genome was previously described as highly recombined (Lorenzi et al. 2022). Comparison to other *att*B sites (Table S3 and S4) reveals that the Samy*-att*B site is located furthest from the origin of replication in most strains (Fig. 2B and C, Fig. S4).

Taken together, these results indicate that the Samy-*att*B site within a gene encoding a radical SAM protein is frequently present across the genus, as a single copy and located in the terminal compartment of the *Streptomyces* chromosome.

### Comparative analysis of the integration via PhiC31- and Samy-based vectors

**Fig. 3:**
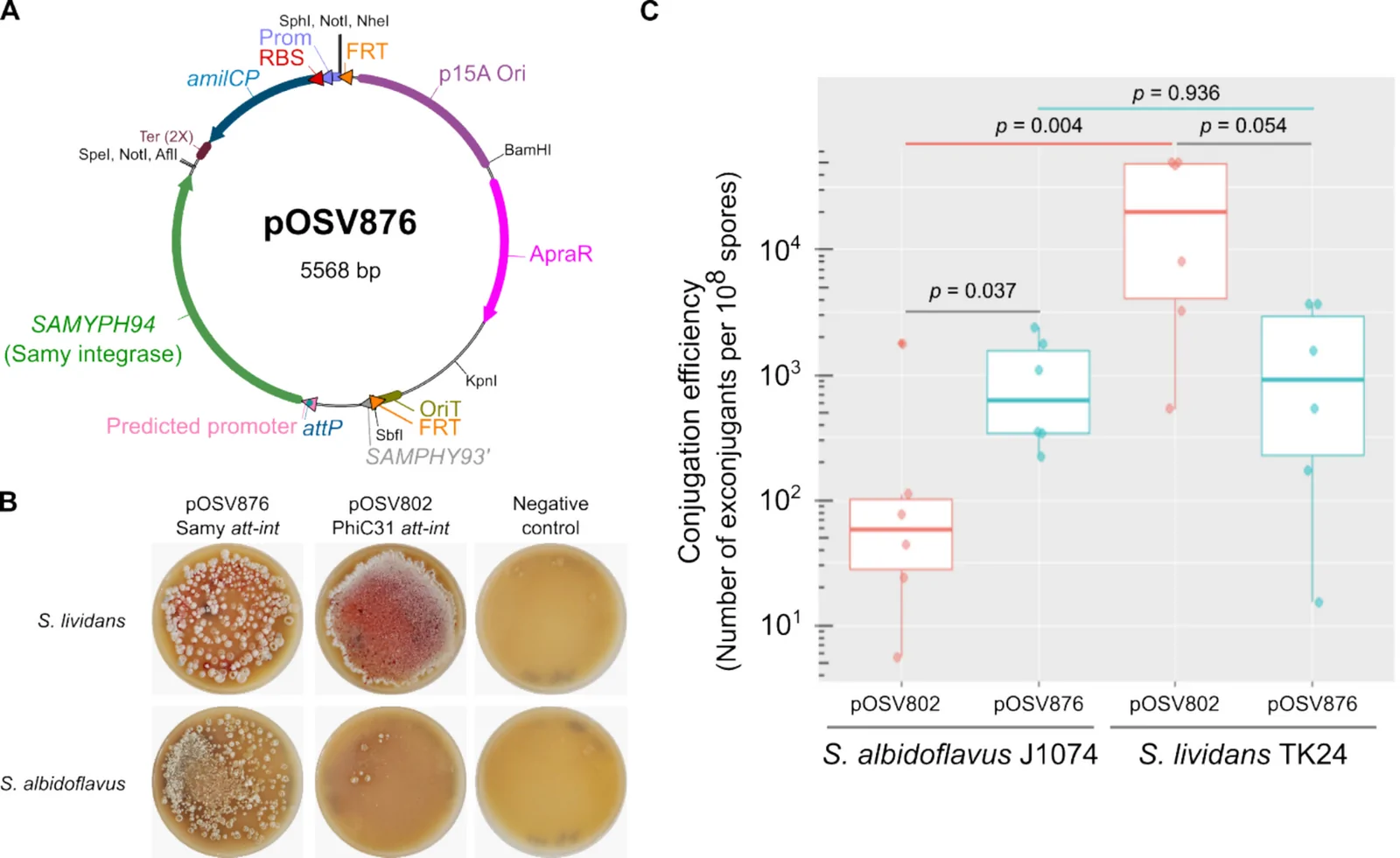
Comparative analysis of Samy- and PhiC31-based integrative systems. **A** Schematic representation of pOSV876 vector. The vector is organized in a modular fashion according to the collection described by Aubry and colleagues (Aubry et al. 2019). The predicted promoter and *attP* site sequences are presented in Figure S1. *amilCP,* gene encoding an *Acropora millepora* blue chromoprotein; ApraR, apramycin resistance; FRT, flippase recognition target; OriT, origin of transfer; p15A Ori, p15A origin of replication in *E. coli*; Prom, *E. coli* promoter; RBS, ribosome binding site; *SAMYPH93’*, fragment of *SAMYPH93* gene; Ter, terminators. **B** A representative result of a conjugation experiment of *S. albidoflavus* J1074 and *S. lividans* TK24 using an *E. coli* strain to transfer pOSV876 and pOSV802. In this latter plasmid, the PhiC31 *att-int* system replaces the Samy system (Aubry et al. 2019). An *E. coli* strain devoid of conjugative plasmid was used as a control. The plates show the exconjugants obtained by spreading a 100-fold dilution from a conjugation experiment conducted with approximately 10^8^ spores. **C** Conjugation efficiency of an integrating vector, containing Samy or PhiC31 *att-int* systems into *S. albidoflavus* J1074 and *S. lividans* TK24. Boxplots are represented as in Fig. 2C. Each dot represents an independent experiment. The *p* values of two-sided Wilcoxon rank sum tests with continuity correction are indicated

The closest known phage to Samy is PhiC31 (Jaffal et al. 2024). However, while Samy integrates within the terminal compartment, PhiC31 typically integrates near the origin of replication in most species (Fig. 2B). To compare the efficiency of integration vectors based on both types of integrases, we introduced the Samy *att*P and integrase encoding gene between SbfI and AflII sites in the pOSV backbone of the plasmid collection built by Aubry and colleagues (Aubry et al. 2019). This allows for the generation of perfectly comparable plasmid vectors, pOSV802 (PhiC31-based) and pOSV876 (Samy-based), that differ only in their integrative modules (Fig. 3A). These vectors were introduced through intergeneric conjugation into the *S. albidoflavus* J1074 and *S. lividans* TK24 strains. The Samy-*att*B sites in these two strains each exhibit 84% identity to the native *att*B sequence in *S. ambofaciens*, though they differ at distinct positions. The efficiency of conjugation by the pOSV876 vector was equivalent—around 10^3^ exconjugants per CFU—between these two host strains, whereas the transduction efficiency of pOSV802 varies by several orders of magnitude between them. Indeed, the pOSV876 conjugation efficiency was one-log higher than that of the pOSV802 vector when the recipient bacteria were *S. albidoflavus* J1074, but lower than that of the pOSV802 vector when the recipient was *S. lividans* TK24 (Fig. 3B and C). This suggests that host-specific factors influence the efficiency of PhiC31 integrative vector-based gene transfer. In particular, the PhiC31-*att*B site in *S. albidoflavus* J1074 shows 91.4% identity to the reference sequence, whereas it is perfectly identical in the *S. lividans* TK24 chromosome (Table S4). This difference may account for all or part of the difference in efficiency between the strains.

Overall, these results suggest that the efficiency of integration and/or expression of the resistance cassette in the terminal compartment using the Samy-based vector is relatively high, comparable to the PhiC31 integrative system, which varies by strain.

### Samy integrase-based vector exhibits a basal level of excision in vivo

**Fig. 4:**
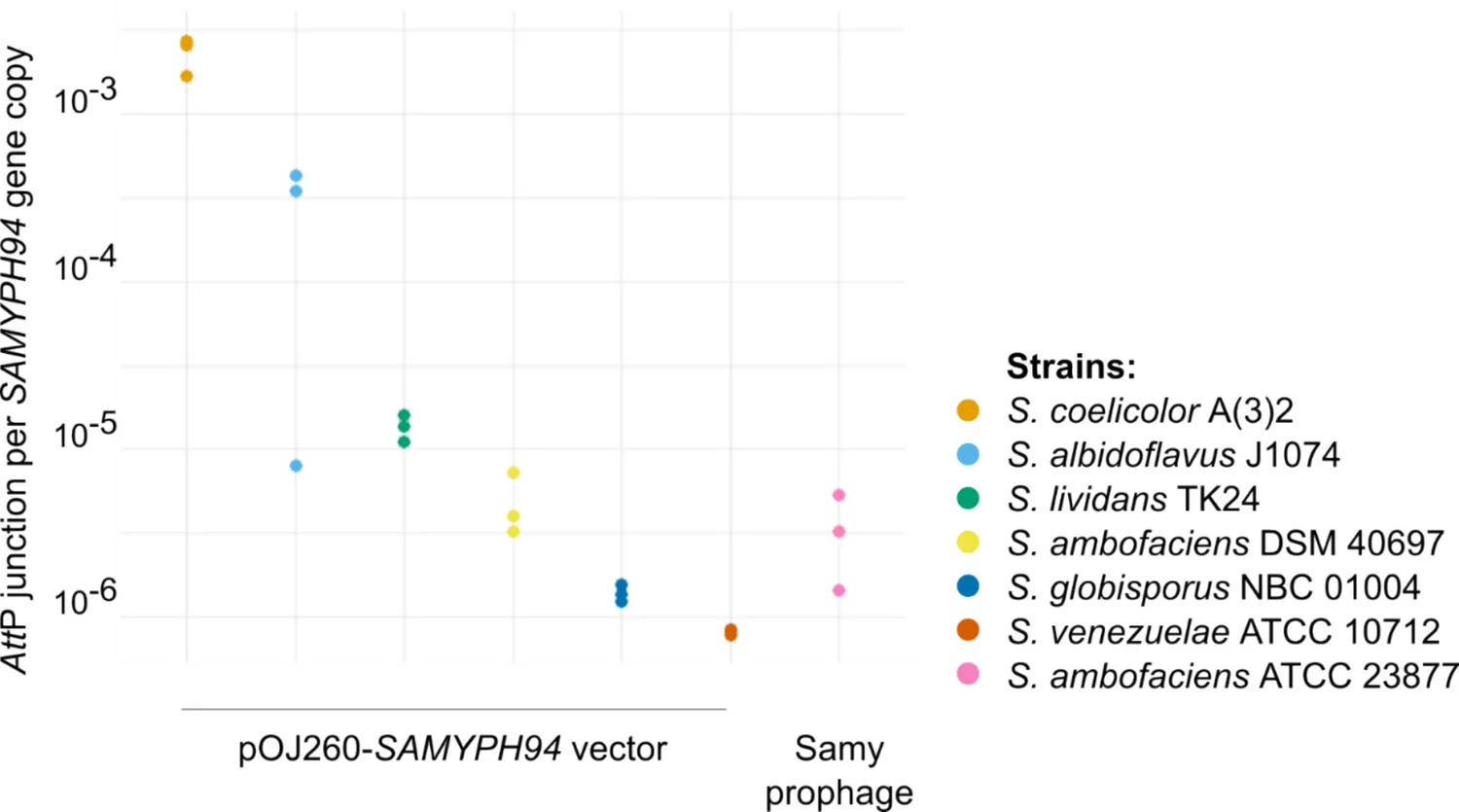
Basal excision level of Samy integrase-based vector and native prophage. The excision levels of the pOJ260_*SAMYPH94* vector and the Samy prophage were assessed by quantitative PCR (qPCR) performed on gDNA from six *Streptomyces* strains in which the pOJ260-*SAMYPH94* vector was integrated, as well as from the native strain containing the complete Samy prophage. For each condition, the *att*P junction copy number was quantified relative to the *SAMYPH94* gene copy number. The results from three quantifications performed on distinct clones are presented

To assess whether Samy integrase-based integration is unidirectional, we quantified the proportion of *att*P junctions in apramycin-resistant exconjugants following the introduction of pOJ260*_SAMYPH94*. Interestingly, the number of *att*P junctions per genome varied between approximately 10⁻⁶ and 10⁻3, depending on the strain, with *S. coelicolor* exhibiting the highest levels of excision (Fig. 4). We further examined this activity within the native context of the Samy prophage in the *S. ambofaciens* ATCC 23877 strain. Notably, we observed a low level of spontaneous excision, comparable to that of pOJ260_*SAMYPH94* in the *S. ambofaciens* DSM 40697 strain, suggesting that the excision activity remains consistent and is not significantly influenced by the presence of other prophage genes.

Upon integration, the *SAMYPH94* gene becomes separated from its native viral promoter—located just upstream of the *att* repeat (Fig. 1B, Fig. S1A). However, on the *att*L side, promoters upstream of the *SAMYPH94* gene have been predicted (Fig. S1B). This finding aligns with previous studies analyzing the Samy transcriptome, which identified *SAMYPH94* as one of the genes consistently expressed across 13 different culture conditions and growth stages (Jaffal et al. 2024).

Altogether, these results suggest that the Samy integrase remains expressed even after integration and may exhibit a basal excisionase activity, influenced by the genetic background and/or host factors. Such an activity has been previously described for other serine-recombinases as PhiJoe (Fogg et al. 2017), PhiBT1 (Zhang et al. 2008), and, more recently, PhiC31 (Duan et al. 2025).

### Heterologous expression of the albonoursin BGC inserted at Samy-, PhiC31-, and R4-*att*B sites in *S. lividans*

To evaluate the efficacy of the Samy integration system for expressing SMBGCs, we replaced the *att-int* PhiC31 module in the previously described pCEA007 plasmid (Aubry et al. 2019)—hereafter referred to as pALB-PhiC31—with the *att-int* Samy module. The resulting pALB-Samy vector therefore corresponds to the pOSV876 vector, in which the albonoursin BGC replaces the *amylCP* module. The albonoursin BGC, first characterized in *S. noursei* (Lautru et al. 2002), consists of three genes—*albA*, *albB*, and *albC*—that are essential for the biosynthesis of albonoursin [cyclo(ΔPhe-ΔLeu)], an antibacterial diketopiperazine containing α,β-dehydro residues. This cluster also directs the production of related cyclodipeptides, including cyclo(Phe-Phe) and the cyclo(Phe-Leu) precursor (Lautru et al. 2002).

For comparative analysis with the only other terminal compartment-targeting system, we also constructed a pALB-R4 derivative using the *att-int* R4 integration system. These vectors—pALB-Samy, pALB-PhiC31, and pALB-R4—were introduced into *S. lividans* TK24 via intergeneric conjugation to assess bacterial fitness and antibiotic production across different genomic integration sites.

We first evaluated the growth rate in MP5 medium of strains carrying pALB vectors inserted into the PhiC31-, R4-, or Samy-*attB* sites by comparing them to the parental TK24 strain lacking the cluster. We found no differences in the generation time of the strains (1.8 h on average) or in the maximal OD reached (Fig. S5). We also assessed the stability of apramycin resistance in these strains after three passages in non-selective medium (Fig. S6), observing no statistically significant differences. These findings indicate that pALB vectors are equally stable and do not compromise strain fitness.

**Fig. 5:**
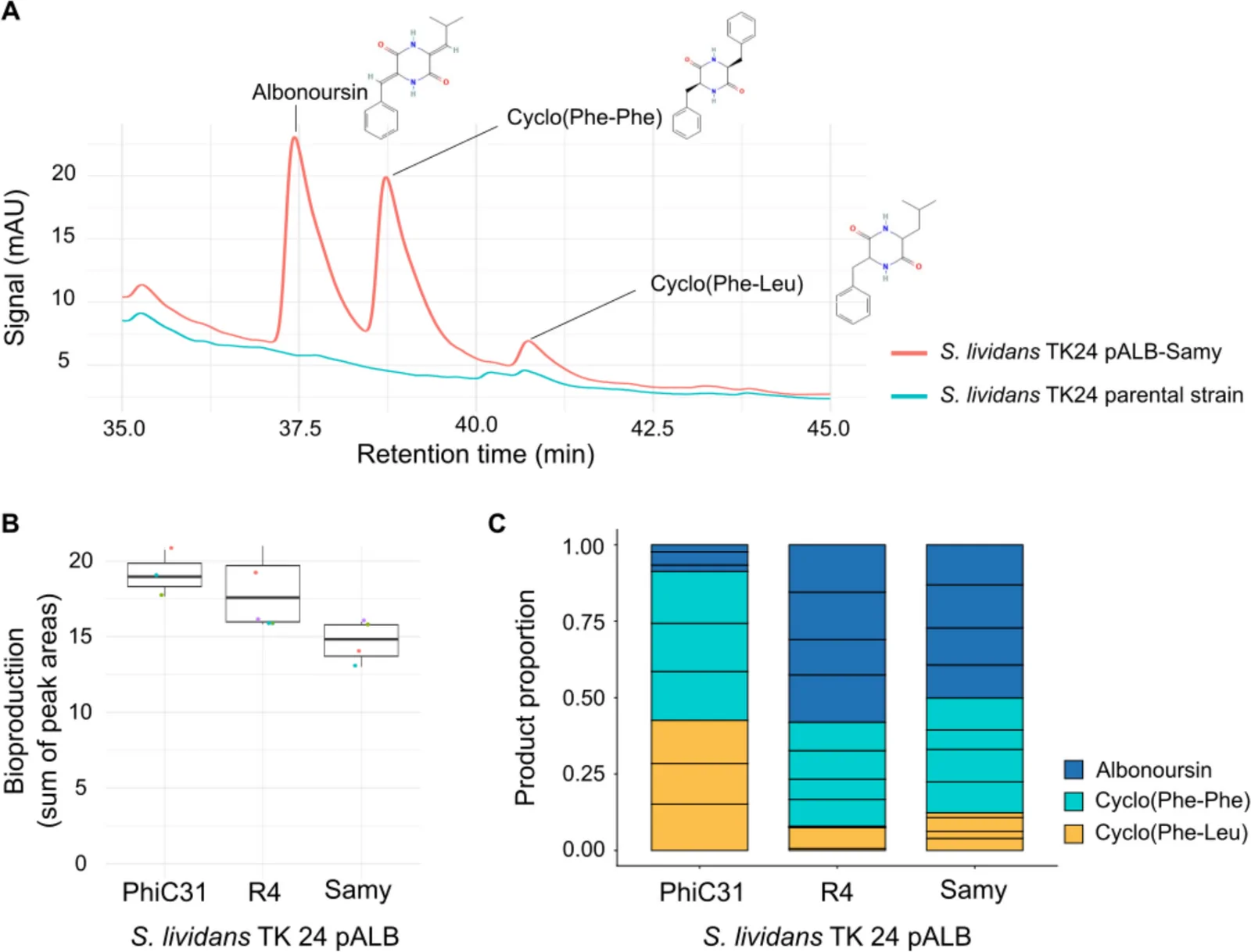
Heterologous expression of the albonoursin BGC inserted at PhiC31-, R4-, or Samy-*att*B sites in *S. lividans*. **A** HPLC analysis of specialized metabolites produced by *S. lividans* pALB-Samy. The chromatogram shows UV signal intensity (milli-Absorbance Units, mAU) at 297 nm for metabolites produced after 4 days of growth in MP5 medium. The positions of albonoursin and related cyclodipeptides (identified by LC-MS; see Fig. S8) are indicated. The supernatant of the parental strain was used as a negative control. Replicates and comparative results for *S. lividans* pALB-PhiC31 and pALB-R4 strains are provided in Figure S7. Molecular formulas for albonoursin, cyclo(Phe-Phe), and cyclo(Phe-Leu) were obtained from NLM PubChem (pubchem.ncbi.nlm.nih.gov). **B** Total production of specialized metabolites by *S. lividans* pALB-PhiC31, pALB-R4, and pALB-Samy. Albonoursin, cyclo(Phe-Phe), and cyclo(Phe-Leu) productions were quantified by HPLC analysis after 4 days of growth in MP5 medium (Fig. S7). The total area under the three peaks was calculated from at least three independent experiments for each of three independent clones per genetic background. Boxplots display the first quartile, median, and third quartile, with whiskers extending to 1.5× the interquartile range (IQR). Each dot represents an independent experiment. No significant differences were detected between strains using pairwise *t*-tests with pooled standard deviation and Bonferroni correction. **C** Proportion of specialized metabolites produced by *S. lividans* harboring the albonoursin BGC at PhiC31-, R4-, or Samy *att*B-sites. The relative proportions of albonoursin, cyclo(Phe-Phe), and cyclo(Phe-Leu) were calculated from HPLC analysis of culture supernatants after 4 days of growth in MP5 medium (Fig. S7). Each sector represents an independent experiment, with data normalized by the number of experiments per strain

Albonoursin production was measured after 4 days of growth in MP5 medium for the *S. lividans* TK24 strains pALB-Samy, pALB-PhiC31, and pALB-R4. *S. noursei* and the parental TK24 strain were used as positive and negative controls, respectively. HPLC and LC-MS analyses revealed that, under these conditions, strains harboring the albonoursin biosynthetic gene cluster produced albonoursin, its cyclodipeptide precursor cyclo(Phe-Leu), as well as cyclo(Phe-Phe) (Fig. 5A, Fig. S7, Fig. S8). Actually, this cluster is expected to produce multiple compounds: AlbC, a prototype of tRNA-dependent cyclodipeptide synthases (CDPS) (Gondry et al. 2009), initiates albonoursin biosynthesis by catalyzing the formation of cyclo(Phe-Leu) from L-Phe-tRNA^Phe^ and L-Leu-tRNA^Leu^ (Lautru et al. 2002). Additionally, AlbC can incorporate other nonpolar residues leading to the production of related cyclodipeptides like cyclo(Phe-Phe) (Gondry et al. 2009). We therefore compared metabolite production across the three strains by summing the yields of albonoursin, cyclo(Phe-Leu), and cyclo(Phe-Phe). No statistically significant differences were observed among the strains with respect to the total production levels of these three compounds (Fig. 5B).

Notably, qualitative differences were detected: strains carrying the cluster at terminal chromosomal locations (Samy or R4) produced higher levels of albonoursin relative to its precursor, whereas the opposite ratio was observed when the cluster was integrated at the ΦC31 site (Fig. 5C). The relative proportions of cyclo(Phe-Phe) were comparable across all strains tested (Fig. 5C). These results indicate that the efficiency of the α,β-dehydrogenation of the two amino acid side chains, catalyzed by the cyclic dipeptide oxidase (CDO) AlbA, with the requirement of AlbB (Lautru et al. 2002), depends on the genomic integration site of the cluster.

Overall, our results show that the Samy integrative tool enables efficient heterologous expression of an SMBGC and that integration at different chromosomal sites can qualitatively influence SMBGC output.

## Discussion

In this study, Samy integration was experimentally validated in six strains and explored using BLAST prediction in a panel of 128 strains. The latter approach has limitations, as some vectors may integrate at pseudo-*att*B sites in the absence of the cognate *att*B site (Combes et al. 2002; Baltz 2012). This capability has been used, for instance, to integrate PhiC31-based tools at the pseudo-*attP* sites in the human genome (Thyagarajan et al. 2001). Conversely, the presence of the *att*B site does not necessarily guarantee an efficient integration, as described for SV1- (Fayed et al. 2014) or PhiWTR- (Ko et al. 2020) based integrative vectors in some strains. The factors involved in the restriction of target cell tropism remain to be elucidated in these cases. Within the abovementioned limits, our bioinformatics analysis indicates that the Samy integrase-based tools enable integration furthest from the origin of replication compared to other integration tools (Fig. 2). Consequently, they extend the range of chromosome regions that can be potentially targeted in *Streptomyces*.

The ideal genetic tool integrates into a “neutral” site, particularly in terms of functions related to fitness and metabolic capacity in *Streptomyces*. This is illustrated by the few tools that restore post-integration the functionality of the target-encoding gene [e.g., µ1/6 (Farkašovská and Godány 2012) and pSAM2 (Boccard et al. 1989)]. However, in most cases, the target gene is inactivated. When this occurs, the impact of integration may not be neutral and requires thorough investigations to be accurately characterized. For instance, integration mediated by PhiC31 has been shown to disturb beta-oxidation metabolism in *S. ambofaciens* ATCC 23877 (Talà et al. 2018). Similarly, integration at the PhiBT1-*att*B site can cause a polar effect on downstream genes, leading to defects in cell differentiation and delayed spore germination (Gonzalez-Quiñonez et al. 2016). Therefore, dedicated experimental studies are crucial to assess the consequences of integration. Samy target gene, *SAM40697_5543*, encodes a radical SAM protein, akin to PhiJoe, PhiOZJ, and PhiWTR phages, which integrate into a distinct radical SAM protein encoding gene (Ko et al. 2020). In some strains, the function of the gene containing Samy-*att*B site is tentatively annotated as potentially involved in DNA repair, based on sequence similarity with SplB. However, this similarity is not strong (Fig. S3), and any connection to DNA repair remains speculative, especially since the surrounding genes at the *att*B site do not appear to be linked to this process. The *SAM40697_5543* gene is inactivated in the environmental strain *S. ambofaciens* ATCC 23877, which is Samy’s native host strain. This suggests that its inactivation does not significantly impact bacterial fitness in nature, consistent with our in vitro observations in this study (Fig. S5 & S6). Moreover, another integrative element is located a few hundred base pairs upstream of Samy*-att*B site, indicating that this region serves as an integration hotspot.

Integrative vectors based on the Samy integrase, like the native virus, exhibit a basal excision level; as assessed by qPCR (Fig. 4). Although considered rare in serine integrases, this bidirectionality has also been reported for PhiJoe (Fogg et al. 2017) and PhiBT1 integrases (Zhang et al. 2008). Fogg and colleagues (Fogg et al. 2017) proposed that the elevated basal excision activity of PhiJoe integrase may result from partial inhibition of synapsis by the coiled-coil motif when the integrase is bound to *att*L and *att*R, reminiscent of the hyperactive PhiC31 mutant IntE449 (Rowley et al. 2008). Most recently, Duan and collaborators developed a sensitive excision detection assay and demonstrated that PhiC31 integrase can also catalyze excision at low frequencies in both *S. lividans* and *E. coli* (Duan et al. 2025). The basal excision level observed with the Samy integrase-derived vector is particularly high in the *S. coelicolor* strain (Fig. 4). This observation suggests that host factors—such as partner proteins and/or the expression level of the integrase in its integrated form—may influence this phenomenon. Interestingly, DNA replication proteins may have been recruited to “moonlight” as RDFs for large serine integrases (Alsaleh et al. 2025). For example, phage TG1 appears to have adapted its integrase to recognize a single-stranded DNA-binding protein as a cofactor for excision (Alsaleh et al. 2025). Moreover, RDFs can exhibit promiscuity, potentially enabling cross-reactivity (MacDonald et al. 2024). These results highlight the need for a more systematic evaluation of the basal excision level of integrative vectors across multiple *Streptomyces* strains. In this study, after three passages without selection pressure, we detected no loss of apramycin resistance conferred by the integrative vector inserted via a Samy-based *att-int* system (Fig. S6). This suggests that Samy-based vectors are not inherently less stable than systems like PhiC31.

Novel integrating vectors are desirable because differences in genome architecture and host factors between strains can limit the effectiveness of existing vectors in specific actinomycetes. Additionally, expanding the repertoire of genetic tools will encourage combinatorial engineering efforts (Li et al. 2017, 2019; Gao and Smith 2021). To complete the suite of *Streptomyces int/attP* vectors, we constructed apramycin-resistant derivatives of pSET152 and pOSV vectors harboring Samy-integrase.

Finally, variations in gene expression depending on their natural chromosomal location (Lioy et al. 2021; Lorenzi et al. 2022) or their integration site (Phelan et al. 2017) further justify the need to enhance the genetic toolbox for *Streptomyces* and other actinomycetes. Naturally, SMBGCs tend to be enriched in the terminal compartments of *Streptomyces* chromosomes (Lioy et al. 2021; Lorenzi et al. 2022). The impact of this localization on their cryptic nature and the strength of their repression or activation in response to environmental conditions remain to be explored. Currently, tools based on Samy and R4 integrases are the only ones targeting the terminal compartment in most strains. Therefore, they are valuable for investigating these questions.

Actually, the albonoursin BGC heterologous expression from vectors integrated at Samy and R4 sites into the terminal chromosomal compartment yields higher albonoursin production compared to integration at the PhiC31-*att*B site. Our findings indicate that these differences stem from variations in the efficiency of the final dehydrogenation step, catalyzed by AlbA with the assistance of AlbB. Since *albA* and *albB* genes are located upstream of *albC*—which encodes the CDPS synthesizing the cyclo(Phe-Leu) precursor—we can exclude issues related to premature operon transcription termination as a site-dependent factor. Differences in redox potential—such as those potentially associated with *pirA* inactivation by insertion at the PhiC31-*att*B site (Talà et al. 2018)—may influence the modification of cyclodipeptides. Overall, these results demonstrate the efficacy of Samy-derived vectors for heterologous expression and highlight the importance of testing multiple integration sites to optimize production of the target compound.

Similarly to pSAM2, the first exploited integrative tool described in *Streptomyces* (Boccard et al. 1989; Raynal et al. 1998), our study demonstrates that integrative systems can be discovered through an approach guided by the study of integrated genetic elements such as prophages. This complements the more traditional method of isolating phages from the environment. Hundreds of complete or defective prophages have been identified in *Streptomyces* genomes (Sharma et al. 2023), highlighting their potential as a valuable source of genetic tools. Exploiting these elements requires the amplification and cloning of their integrases and putative *attP* sites, as predicted by identifying the repeats bordering the prophages. To achieve this, the conditions that activate the efficient excision of these integrated mobile genetic elements have to be identified, as we did earlier for the Samy prophage (Jaffal et al. 2024). However, this may constitute a bottleneck for exploiting these systems. Alternatively, artificial excision can be used to obtain circular prophage form. This is required for simultaneously cloning the integrase and the predicted excision factor. With this approach, the cloning of the excision tool is a prerequisite for exploiting the integrative potential of prophages and integrative mobile genetic elements. Finally, synthetic biology provides the option to directly synthesize genes encoding integrases and their predicted *att*P sites. Collectively, these different strategies offer options for expanding the toolbox for precisely integrating and excising (over)cassettes into genomes.

## Supplementary Information

Below is the link to the electronic supplementary material.

ESM1 (ZIP 20.6 MB)

## Data Availability

The* att*B sequences used in this study are detailed in Fig.  1 C and Table S3. The bioinformatics analyses supporting some of the figures are summarized in Table S4. The integrative vectors generated in this study are available upon request from the corresponding author (SBM).

## References

[CR1] Abramson J, Adler J, Dunger J, Evans R, Green T, Pritzel A, Ronneberger O, Willmore L, Ballard AJ, Bambrick J, Bodenstein SW, Evans DA, Hung C-C, O’Neill M, Reiman D, Tunyasuvunakool K, Wu Z, Žemgulytė A, Arvaniti E, Beattie C, Bertolli O, Bridgland A, Cherepanov A, Congreve M, Cowen-Rivers AI, Cowie A, Figurnov M, Fuchs FB, Gladman H, Jain R, Khan YA, Low CMR, Perlin K, Potapenko A, Savy P, Singh S, Stecula A, Thillaisundaram A, Tong C, Yakneen S, Zhong ED, Zielinski M, Žídek A, Bapst V, Kohli P, Jaderberg M, Hassabis D, Jumper JM (2024) Accurate structure prediction of biomolecular interactions with AlphaFold 3. Nature 630:493–500. 10.1038/s41586-024-07487-w 38718835 10.1038/s41586-024-07487-w PMC11168924

[CR2] Alsaleh A, Holland A, Shin H, Reyes TP, Baksh A, Taiwo-Aiyerin OT, Pigli Y, Rice PA, Olorunniji FJ (2025) Large serine integrases utilise scavenged phage proteins as directionality cofactors. Nucleic Acids Res 53:gkaf050. 10.1093/nar/gkaf050 39907112 10.1093/nar/gkaf050 PMC11795197

[CR3] Aubry C, Pernodet JL, Lautru S (2019) Modular and integrative vectors for synthetic biology applications in *Streptomyces* spp. Appl Environ Microbiol 85. 10.1128/AEM.00485-19 10.1128/AEM.00485-19 PMC6677859 31175189

[CR4] Ba F, Zhang Y, Wang L, Liu W-Q, Li J (2023) Applications of serine integrases in synthetic biology over the past decade. SynBio 1:172–189. 10.3390/synbio1020012

[CR5] Baltz RH (2012) *Streptomyces* temperate bacteriophage integration systems for stable genetic engineering of actinomycetes (and other organisms). J Ind Microbiol Biotechnol 39:661–672. 10.1007/s10295-011-1069-6 22160317 10.1007/s10295-011-1069-6

[CR6] Bierman M, Logan R, O’Brien K, Seno ET, Rao RN, Schoner BE (1992) Plasmid cloning vectors for the conjugal transfer of DNA from *Escherichia coli* to *Streptomyces* spp. Gene 116:43–49 1628843 10.1016/0378-1119(92)90627-2

[CR7] Boccard F, Smokvina T, Pernodet JL, Friedmann A, Guérineau M (1989) The integrated conjugative plasmid pSAM2 of *Streptomyces ambofaciens* is related to temperate bacteriophages. EMBO J 8:973–980. 10.1002/j.1460-2075.1989.tb03460.x 2721504 10.1002/j.1460-2075.1989.tb03460.x PMC400899

[CR8] Bury-Moné S, Thibessard A, Lioy VS, Leblond P (2023) Dynamics of the *Streptomyces* chromosome: chance and necessity. Trends Genet. 10.1016/j.tig.2023.07.008 37679290 10.1016/j.tig.2023.07.008

[CR9] Choufa C, Tidjani A-R, Gauthier A, Harb M, Lao J, Leblond-Bourget N, Vos M, Leblond P, Bontemps C (2022) Prevalence and mobility of integrative and conjugative elements within a *Streptomyces* natural population. Front Microbiol 13:970179. 10.3389/fmicb.2022.970179 36177458 10.3389/fmicb.2022.970179 PMC9513070

[CR10] Combes P, Till R, Bee S, Smith MCM (2002) The *Streptomyces* genome contains multiple pseudo- *attB* sites for the φC31-encoded site-specific recombination system. J Bacteriol 184:5746–5752. 10.1128/JB.184.20.5746-5752.2002 12270833 10.1128/JB.184.20.5746-5752.2002 PMC139614

[CR11] Crooks GE, Hon G, Chandonia J-M, Brenner SE (2004) WebLogo: a sequence logo generator. Genome Res 14:1188–1190. 10.1101/gr.849004 15173120 10.1101/gr.849004 PMC419797

[CR12] Deng L, Zhao Z, Liu L, Zhong Z, Xie W, Zhou F, Xu W, Zhang Y, Deng Z, Sun Y (2023) Dissection of 3d chromosome organization in *Streptomyces coelicolor* A3(2) leads to biosynthetic gene cluster overexpression. Proc Natl Acad Sci U S A 120:e2222045120. 10.1073/pnas.2222045120 36877856 10.1073/pnas.2222045120 PMC10242723

[CR14] Di Salvo M, Pinatel E, Talà A, Fondi M, Peano C, Alifano P (2018) G4promfinder: an algorithm for predicting transcription promoters in GC-rich bacterial genomes based on AT-rich elements and G-quadruplex motifs. BMC Bioinformatics 19:36. 10.1186/s12859-018-2049-x 29409441 10.1186/s12859-018-2049-x PMC5801747

[CR15] Dong M-J, Luo H, Gao F (2022) Ori-finder 2022: a comprehensive web server for prediction and analysis of bacterial replication origins. Genomics Proteomics Bioinformatics 20:1207–1213. 10.1016/j.gpb.2022.10.002 36257484 10.1016/j.gpb.2022.10.002 PMC10225481

[CR16] Duan Y, Liu Z, Huang X, Xu L, Wang X, Liu H, Xie Z (2025) Mitigating genetic instability caused by the excision activity of the *phi* C31 integrase in *Streptomyces*. Appl Environ Microbiol 91:e01812-24. 10.1128/aem.01812-24 39704534 10.1128/aem.01812-24 PMC11784100

[CR17] Farkašovská J, Godány A (2012) Analysis of the site-specific integration system of the *Streptomyces aureofaciens* phage μ1/6. Curr Microbiol 64:226–233. 10.1007/s00284-011-0054-7 22143397 10.1007/s00284-011-0054-7

[CR18] Fayed B, Younger E, Taylor G, Smith MCM (2014) A novel *Streptomyces* spp. integration vector derived from the *S. venezuelae* phage, SV1. BMC Biotechnol 14:51. 10.1186/1472-6750-14-51 24885867 10.1186/1472-6750-14-51 PMC4068962

[CR19] Fogg PCM, Haley JA, Stark WM, Smith MCM (2017) Genome integration and excision by a new *Streptomyces* bacteriophage, ϕJoe. Appl Environ Microbiol 83:e02767-16. 10.1128/AEM.02767-16 10.1128/AEM.02767-16 PMC5311408 28003200

[CR20] Gao H, Smith MCM (2021) Use of orthogonal serine integrases to multiplex plasmid conjugation and integration from *E. coli* into *Streptomyces*. Access Microbiol 3. 10.1099/acmi.0.000291 10.1099/acmi.0.000291 PMC8749152 35024553

[CR21] Gao H, Murugesan B, Hoßbach J, Evans SK, Stark WM, Smith MCM (2019) Integrating vectors for genetic studies in the rare Actinomycete *Amycolatopsis marina*. BMC Biotechnol 19:32. 10.1186/s12896-019-0521-y 31164159 10.1186/s12896-019-0521-y PMC6549336

[CR22] Gondry M, Sauguet L, Belin P, Thai R, Amouroux R, Tellier C, Tuphile K, Jacquet M, Braud S, Courçon M, Masson C, Dubois S, Lautru S, Lecoq A, Hashimoto S, Genet R, Pernodet J-L (2009) Cyclodipeptide synthases are a family of tRNA-dependent peptide bond–forming enzymes. Nat Chem Biol 5:414–420. 10.1038/nchembio.175 19430487 10.1038/nchembio.175

[CR23] Gonzalez-Quiñonez N, López-García MT, Yagüe P, Rioseras B, Pisciotta A, Alduina R, Manteca Á (2016) New ΦBT1 site-specific integrative vectors with neutral phenotype in *Streptomyces*. Appl Microbiol Biotechnol 100:2797–2808. 10.1007/s00253-015-7271-0 26758297 10.1007/s00253-015-7271-0

[CR24] Gregory MA, Till R, Smith MCM (2003) Integration site for *Streptomyces* phage φBT1 and development of site-specific integrating vectors. J Bacteriol 185:5320–5323. 10.1128/JB.185.17.5320-5323.2003 12923110 10.1128/JB.185.17.5320-5323.2003 PMC180994

[CR25] Groth AC, Olivares EC, Thyagarajan B, Calos MP (2000) A phage integrase directs efficient site-specific integration in human cells. Proc Natl Acad Sci U S A 97:5995–6000. 10.1073/pnas.090527097 10801973 10.1073/pnas.090527097 PMC18547

[CR26] Hou Y, Zhang H, Miranda L, Lin S (2010) Serious overestimation in quantitative PCR by circular (supercoiled) plasmid standard: microalgal pcna as the model gene. PLoS ONE 5:e9545. 10.1371/journal.pone.0009545 20221433 10.1371/journal.pone.0009545 PMC2832698

[CR27] Jaffal H, Kortebi M, Misson P, Tavares P, Ouldali M, Leh H, Lautru S, Lioy VS, Lecointe F, Bury-Moné SG (2024) Prophage induction can facilitate the *in vitro* dispersal of multicellular *Streptomyces* structures. PLoS Biol 22:e3002725. 10.1371/journal.pbio.3002725 39052683 10.1371/journal.pbio.3002725 PMC11302927

[CR28] Kieser T, Buttner MJ, Chater KF, Hopwood DA (2000) Practical *Streptomyces* genetics. John Innes Foundation, Norwich, United Kingdom

[CR29] Ko B, D’Alessandro J, Douangkeomany L, Stumpf S, deButts A, Blodgett J (2020) Construction of a new integrating vector from actinophage ϕOZJ and its use in multiplex *Streptomyces* transformation. J Ind Microbiol Biotechnol 47:73–81. 10.1007/s10295-019-02246-7 31705217 10.1007/s10295-019-02246-7

[CR30] Kormanec J, Rezuchova B, Homerova D, Csolleiova D, Sevcikova B, Novakova R, Feckova L (2019) Recent achievements in the generation of stable genome alterations/mutations in species of the genus *Streptomyces*. Appl Microbiol Biotechnol 103:5463–5482. 10.1007/s00253-019-09901-0 31119353 10.1007/s00253-019-09901-0

[CR31] Lautru S, Gondry M, Genet R, Pernodet J-L (2002) The albonoursin gene cluster of *S. noursei*. Chem Biol 9:1355–1364. 10.1016/S1074-5521(02)00285-5 12498889 10.1016/s1074-5521(02)00285-5

[CR32] Lee N, Hwang S, Lee Y, Cho S, Palsson B, Cho B-K (2019) Synthetic biology tools for novel secondary metabolite discovery in *Streptomyces*. J Microbiol Biotechnol 29:667–686. 10.4014/jmb.1904.04015 31091862 10.4014/jmb.1904.04015

[CR33] Li L, Zheng G, Chen J, Ge M, Jiang W, Lu Y (2017) Multiplexed site-specific genome engineering for overproducing bioactive secondary metabolites in actinomycetes. Metab Eng 40:80–92. 10.1016/j.ymben.2017.01.004 28088540 10.1016/j.ymben.2017.01.004

[CR34] Li L, Wei K, Liu X, Wu Y, Zheng G, Chen S, Jiang W, Lu Y (2019) aMSGE: advanced multiplex site-specific genome engineering with orthogonal modular recombinases in actinomycetes. Metab Eng 52:153–167. 10.1016/j.ymben.2018.12.001 30529239 10.1016/j.ymben.2018.12.001

[CR35] Lioy VS, Lorenzi J-N, Najah S, Poinsignon T, Leh H, Saulnier C, Aigle B, Lautru S, Thibessard A, Lespinet O, Leblond P, Jaszczyszyn Y, Gorrichon K, Varoquaux N, Junier I, Boccard F, Pernodet J-L, Bury-Moné S (2021) Dynamics of the compartmentalized *Streptomyces* chromosome during metabolic differentiation. Nat Commun 12:5221. 10.1038/s41467-021-25462-1 34471117 10.1038/s41467-021-25462-1 PMC8410849

[CR36] Lorenzi J-N, Thibessard A, Lioy VS, Boccard F, Leblond P, Pernodet J-L, Bury-Moné S (2022) Ribosomal RNA operons define a central functional compartment in the *Streptomyces* chromosome. Nucleic Acids Res 50:11654–11669. 10.1093/nar/gkac1076 36408918 10.1093/nar/gkac1076 PMC9723626

[CR37] MacDonald AI, Baksh A, Holland A, Shin H, Rice PA, Stark WM, Olorunniji FJ (2024) Variable orthogonality of serine integrase interactions within the ϕC31 family. Sci Rep 14:26280. 10.1038/s41598-024-77570-9 39487291 10.1038/s41598-024-77570-9 PMC11530663

[CR38] McDonald BR, Currie CR (2017) Lateral gene transfer dynamics in the ancient bacterial genus *Streptomyces*. Mbio. 10.1128/mBio.00644-17 28588130 10.1128/mBio.00644-17 PMC5472806

[CR39] Merrick CA, Zhao J, Rosser SJ (2018) Serine integrases: advancing synthetic biology. ACS Synth Biol 7:299–310. 10.1021/acssynbio.7b00308 29316791 10.1021/acssynbio.7b00308

[CR40] Mitousis L, Thoma Y, Musiol-Kroll EM (2020) An update on molecular tools for genetic engineering of actinomycetes—the source of important antibiotics and other valuable compounds. Antibiotics 9:494. 10.3390/antibiotics9080494 32784409 10.3390/antibiotics9080494 PMC7460540

[CR41] Miura T, Hosaka Y, Yan-Zhuo Y, Nishizawa T, Asayama M, Takahashi H, Shirai M (2011) *In vivo* and *in vitro* characterization of site-specific recombination of actinophage R4 integrase. J Gen Appl Microbiol 57:45–57. 10.2323/jgam.57.45 21478647 10.2323/jgam.57.45

[CR42] Morita K, Yamamoto T, Fusada N, Komatsu M, Ikeda H, Hirano N, Takahashi H (2009) The site-specific recombination system of actinophage TG1. FEMS Microbiol Lett 297:234–240. 10.1111/j.1574-6968.2009.01683.x 19624407 10.1111/j.1574-6968.2009.01683.x

[CR43] Oberto J (2013) Synttax: a web server linking synteny to prokaryotic taxonomy. BMC Bioinformatics 14:4. 10.1186/1471-2105-14-4 23323735 10.1186/1471-2105-14-4 PMC3571937

[CR44] Pernodet JL, Alegre MT, Blondelet-Rouault MH, Guerineau M (1993) Resistance to spiramycin in *Streptomyces ambofaciens*, the producer organism, involves at least two different mechanisms. J Gen Microbiol 139:1003–1011. 10.1099/00221287-139-5-1003 7687646 10.1099/00221287-139-5-1003

[CR45] Phelan RM, Sachs D, Petkiewicz SJ, Barajas JF, Blake-Hedges JM, Thompson MG, Reider Apel A, Rasor BJ, Katz L, Keasling JD (2017) Development of next generation synthetic biology tools for use in *Streptomyces venezuelae*. ACS Synth Biol 6:159–166. 10.1021/acssynbio.6b00202 27605473 10.1021/acssynbio.6b00202

[CR46] R Core Team (2023) R: a language and environment for statistical computing. R Foundation for Statistical Computing. Vienna, Austria. https://www.R-project.org/

[CR47] Rangannan V, Bansal M (2009) Relative stability of DNA as a generic criterion for promoter prediction: whole genome annotation of microbial genomes with varying nucleotide base composition. Mol Biosyst 5:1758. 10.1039/b906535k 19593472 10.1039/B906535K

[CR48] Raynal A, Tuphile K, Gerbaud C, Luther T, Guérineau M, Pernodet J (1998) Structure of the chromosomal insertion site for pSAM2: functional analysis in *Escherichia coli*. Mol Microbiol 28:333–342. 10.1046/j.1365-2958.1998.00799.x 9622358 10.1046/j.1365-2958.1998.00799.x

[CR49] Rowley PA, Smith MCA, Younger E, Smith MCM (2008) A motif in the C-terminal domain of ϕC31 integrase controls the directionality of recombination. Nucleic Acids Res 36:3879–3891. 10.1093/nar/gkn269 18502775 10.1093/nar/gkn269 PMC2475636

[CR50] Sharma V, Hünnefeld M, Luthe T, Frunzke J (2023) Systematic analysis of prophage elements in actinobacterial genomes reveals a remarkable phylogenetic diversity. Sci Rep 13:4410. 10.1038/s41598-023-30829-z 36932119 10.1038/s41598-023-30829-z PMC10023795

[CR51] Slieman TA, Rebeil R, Nicholson WL (2000) Spore photoproduct (SP) lyase from *Bacillus subtilis* specifically binds to and cleaves SP (5-Thyminyl-5,6-Dihydrothymine) but not cyclobutane pyrimidine dimers in UV-irradiated DNA. J Bacteriol 182:6412–6417. 10.1128/JB.182.22.6412-6417.2000 11053385 10.1128/jb.182.22.6412-6417.2000 PMC94787

[CR52] Smith MCM (2015) Phage-encoded serine integrases and other large serine recombinases. Microbiol Spectr 3:3.4.06. 10.1128/microbiolspec.MDNA3-0059-2014 10.1128/microbiolspec.MDNA3-0059-2014 26350324

[CR53] Szafran MJ, Małecki T, Strzałka A, Pawlikiewicz K, Duława J, Zarek A, Kois-Ostrowska A, Findlay KC, Le TBK, Jakimowicz D (2021) Spatial rearrangement of the *Streptomyces venezuelae* linear chromosome during sporogenic development. Nat Commun 12:5222. 10.1038/s41467-021-25461-2 34471115 10.1038/s41467-021-25461-2 PMC8410768

[CR54] Talà A, Damiano F, Gallo G, Pinatel E, Calcagnile M, Testini M, Fico D, Rizzo D, Sutera A, Renzone G, Scaloni A, De Bellis G, Siculella L, De Benedetto GE, Puglia AM, Peano C, Alifano P (2018) Pirin: a novel redox-sensitive modulator of primary and secondary metabolism in *Streptomyces*. Metab Eng 48:254–268. 10.1016/j.ymben.2018.06.008 29944936 10.1016/j.ymben.2018.06.008

[CR55] Thyagarajan B, Olivares EC, Hollis RP, Ginsburg DS, Calos MP (2001) Site-specific genomic integration in mammalian cells mediated by phage φC31 integrase. Mol Cell Biol 21:3926–3934. 10.1128/MCB.21.12.3926-3934.2001 11359900 10.1128/MCB.21.12.3926-3934.2001 PMC87055

[CR56] Van Bergeijk DA, Terlouw BR, Medema MH, Van Wezel GP (2020) Ecology and genomics of Actinobacteria: new concepts for natural product discovery. Nat Rev Microbiol 18:546–558. 10.1038/s41579-020-0379-y 32483324 10.1038/s41579-020-0379-y

[CR57] Van Mellaert L, Mei L, Lammertyn E, Schacht S, Ann J (1998) Site-specific integration of bacteriophage VWB genome into *Streptomyces venezuelae* and construction of a VWB-based integrative vector. Microbiology 144:3351–3358. 10.1099/00221287-144-12-3351 9884227 10.1099/00221287-144-12-3351

[CR58] Zhang L, Ou X, Zhao G, Ding X (2008) Highly efficient *in vitro* site-specific recombination system based on *Streptomyces* phage φBT1 integrase. J Bacteriol 190:6392–6397. 10.1128/JB.00777-08 18689469 10.1128/JB.00777-08 PMC2565996

