# Supplementary material for: A new-engineered integrative tool to target the terminal compartment of the Streptomyces chromosome
Source: Appl Microbiol Biotechnol. 2026 Feb 21;110(1):73. doi: 10.1007/s00253-026-13707-2 (PMC12924846; doi:10.1007/s00253-026-13707-2)
Supplement: Supplementary file 1 — (ZIP 20.6 MB) [file 253_2026_13707_MOESM1_ESM.zip › Samy_integration_system_supp_data_AMB_R2.pdf]

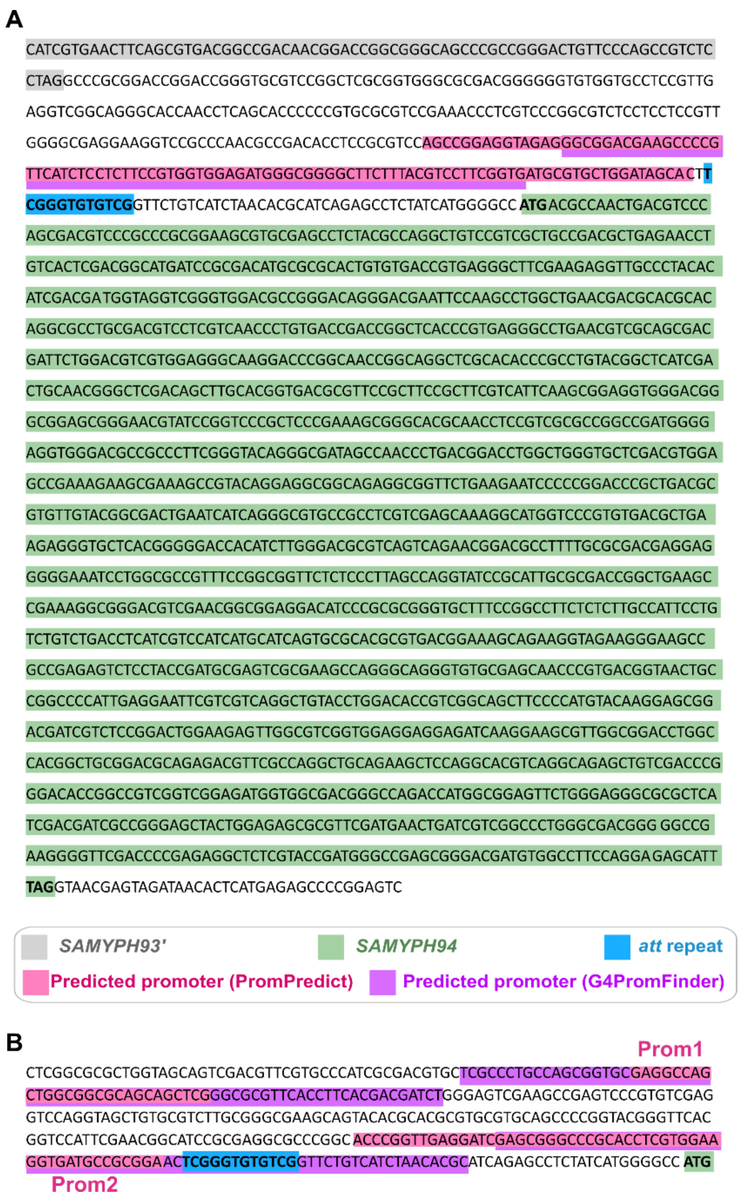

Figure S1: Samy integrase gene and its upstream sequences

A. Sequence cloned in pOJ260_*SAMYPH94* and pOSV876 vectors. The promoter predicted with a confidence level of 4 using PromPredict software (version 1, (Rangannan and Bansal 2009)) overlaps with the promoter predicted by G4PromFinder (Di Salvo 2017; Di Salvo et al. 2018). Start and stop codons as well as the repeat present in *att* sites (**Table 2**) are written in bold. The color code is indicated below the sequence.

B. Sequence upstream *SAMYPH94* gene after integration at the *S. ambofaciens att*B site. The region corresponds to the *att*L side shared by the ATCC 23877 and DSM 40697 strains. The promoters 'Prom1' and 'Prom2'O were predicted on the ATCC 23877 strain by the PromPredict software (version 1, (Rangannan and Bansal 2009)) with confidence levels of 2 and 3, respectively. They both overlap with the promoters predicted by G4PromFinder (Di Salvo 2017; Di Salvo et al. 2018). The color coding follows the scheme used in panel A. Only the start codon of *SAMYPH94* is indicated.

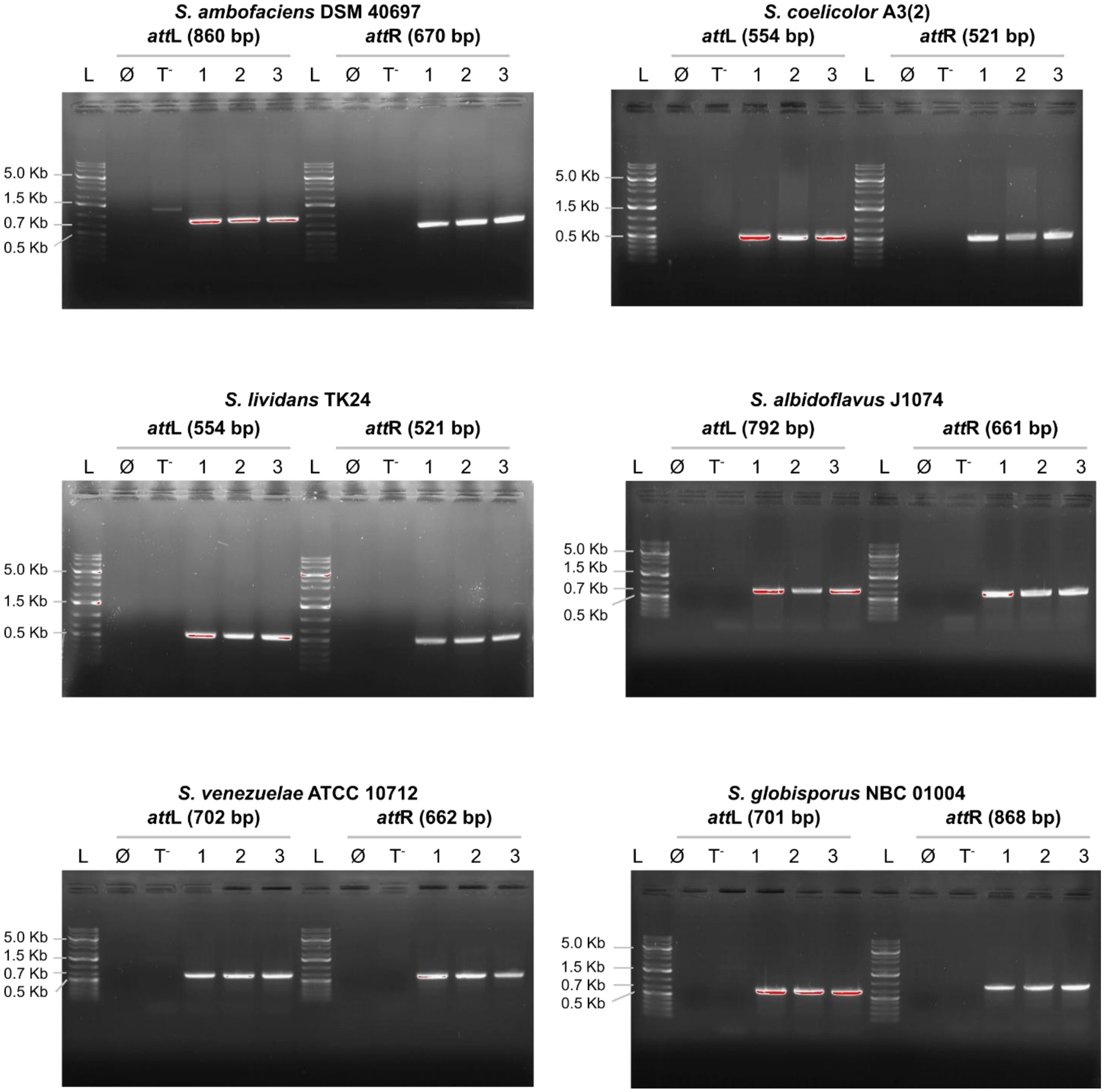

Figure S2: Analysis of the integration site in three independent clones per strain of interest

As described in **Fig 1B,** PCR amplification of the *att*L and *att*R regions defined based on Samy prophage orientation in its native *S. ambofaciens* ATCC 23877 host are shown, with expected sizes indicated in brackets. No PCR amplification is expected when genomic DNA from wild-type *Streptomyces* strains is used as the template ('T-') or in absence of template ('∅'). The numbers represent distinct clones analyzed for each strain. 'L' denotes the molecular weight ladder (Thermo Scientific GeneRuler DNA Ladder 1Kb Plus).

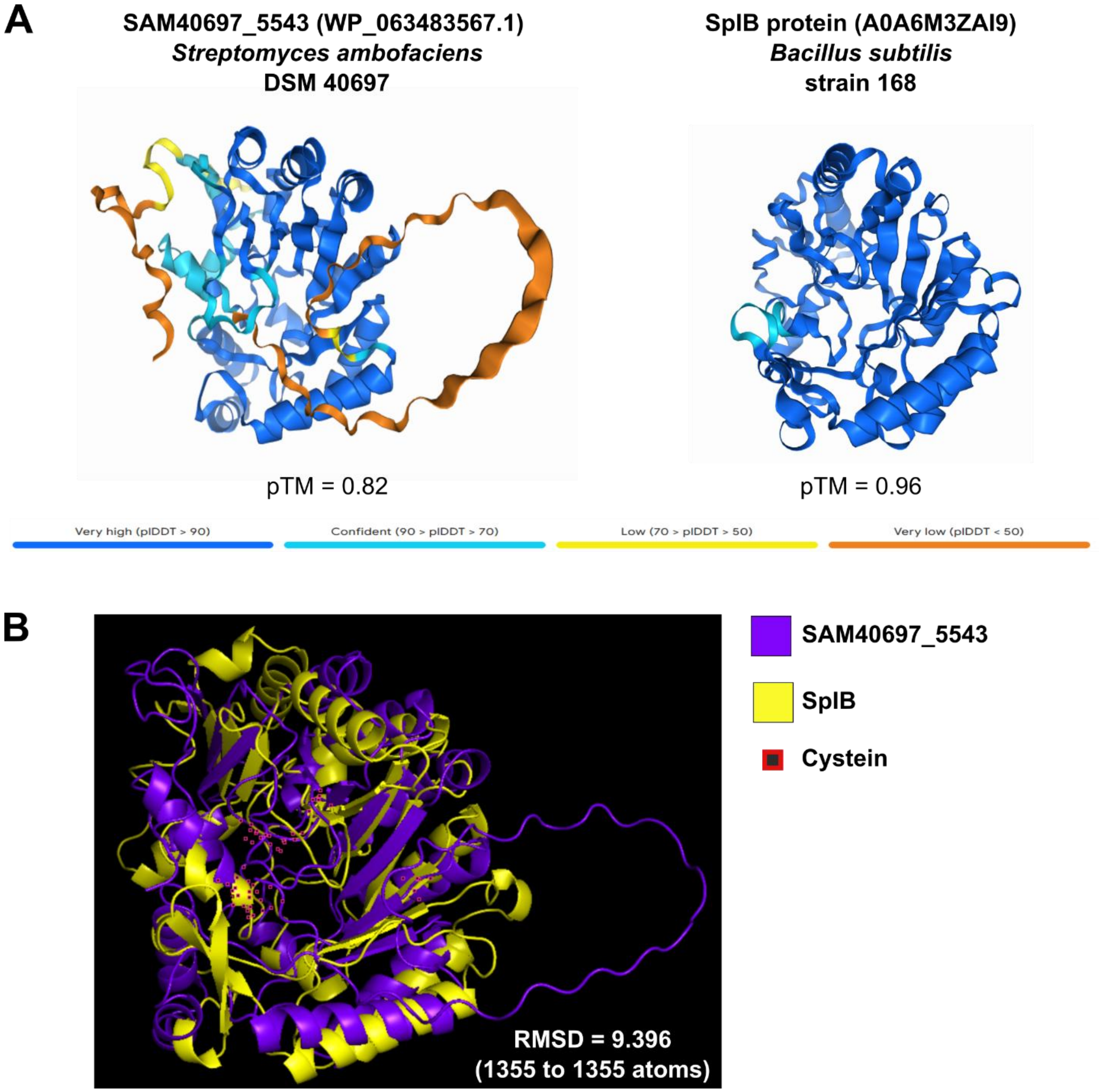

Figure S3: Comparative analysis of the AlphaFold 3 predicted structures of SAM40697_5543 from *S. ambofaciens* DSM 40697 and SplB from *B. subtilis* strain 168

Structures are shown individually in panel A and overlaid in panel B. The RMSD (root-mean-square deviation of atomic positions) was calculated by PyMOL (v.2.6.0a0). The number of positions taken into account for the calculation is indicated in each case. AlphaFold produced a per-residue model confidence score (pLDDT) between 0 and 100. Some regions below 50 pLDDT may be unstructured in isolation.

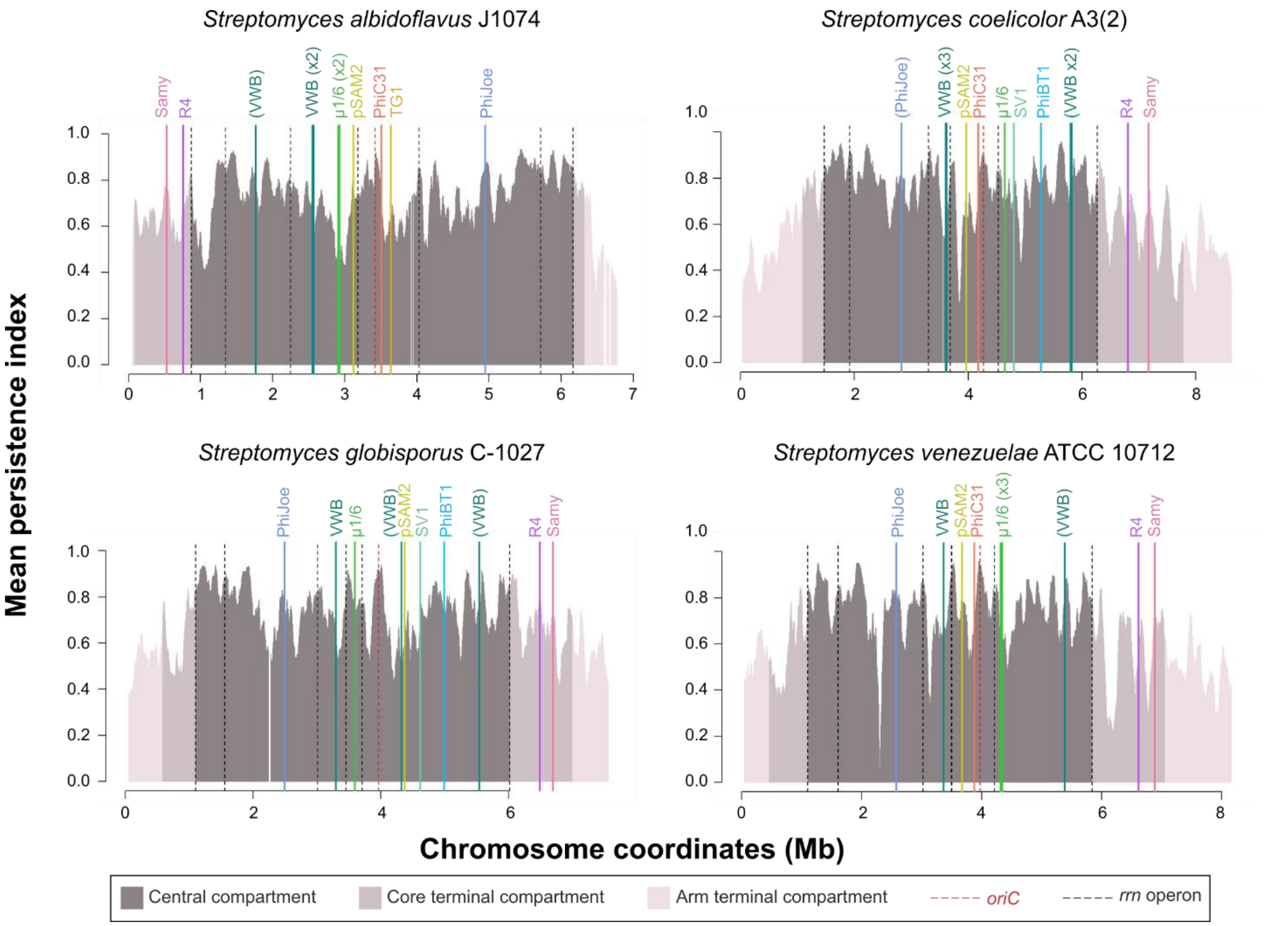

Figure S4: Chromosomal locations of *attB* sites for all *att-int* systems identified to date for engineering the *Streptomyces* chromosome in four strains of interest

The level of gene persistence along the chromosome obtained from (Lorenzi et al. 2022) is represented using a sliding window (81 coding sequences (CDSs), with 1 CDS steps). The positions of all *rrn* operons and of the origin of replication are indicated by dashed black and red lines, respectively. The central compartment (dark gray) is delimitated by the distal *rrn*. Terminal compartments are highlighted in dark pink when they include core genome genes, and in light pink when they do not. Sites that deviate from the consensus sequence (coverage < 80%) are shown in brackets. Numbers indicate the frequency of site occurrence.

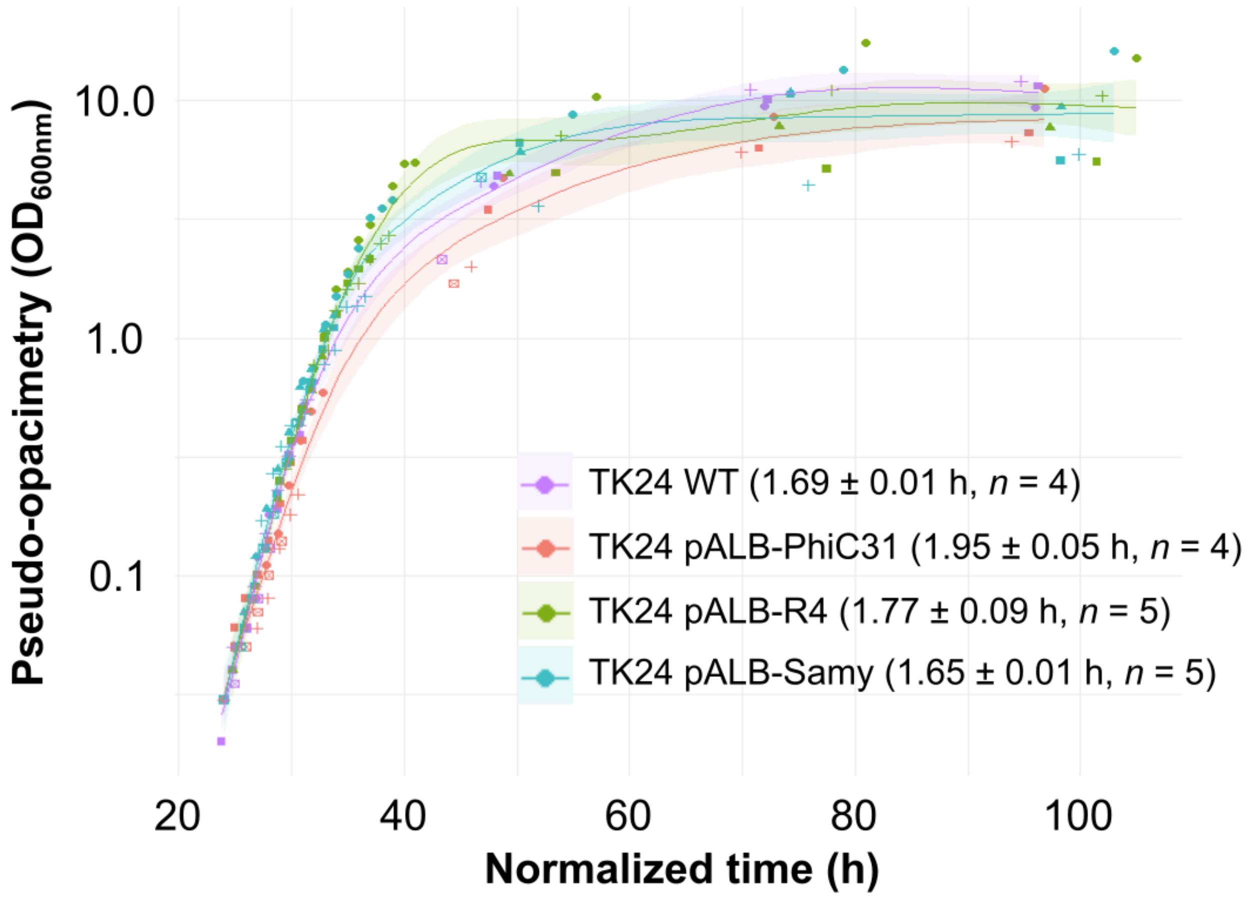

Figure S5: Growth curves of *S. lividans* TK24 strains harboring the albonoursin BGC inserted at PhiC31-, R4- or Samy-*att*B sites

Each point shape represents data from independent experiments (*n* = 4–5 per strain), using three independent clones per strain. Measurements were normalized over a 24 h latency phase. A trend line was fitted using the Generalized Additive Model (GAM) method. The generation time is indicated as mean ± standard error (*n* = number of values). No significant differences were identified in pairwise comparisons using the Wilcoxon rank-sum test with continuity correction and Benjamini–Hochberg adjusted *p*-values.

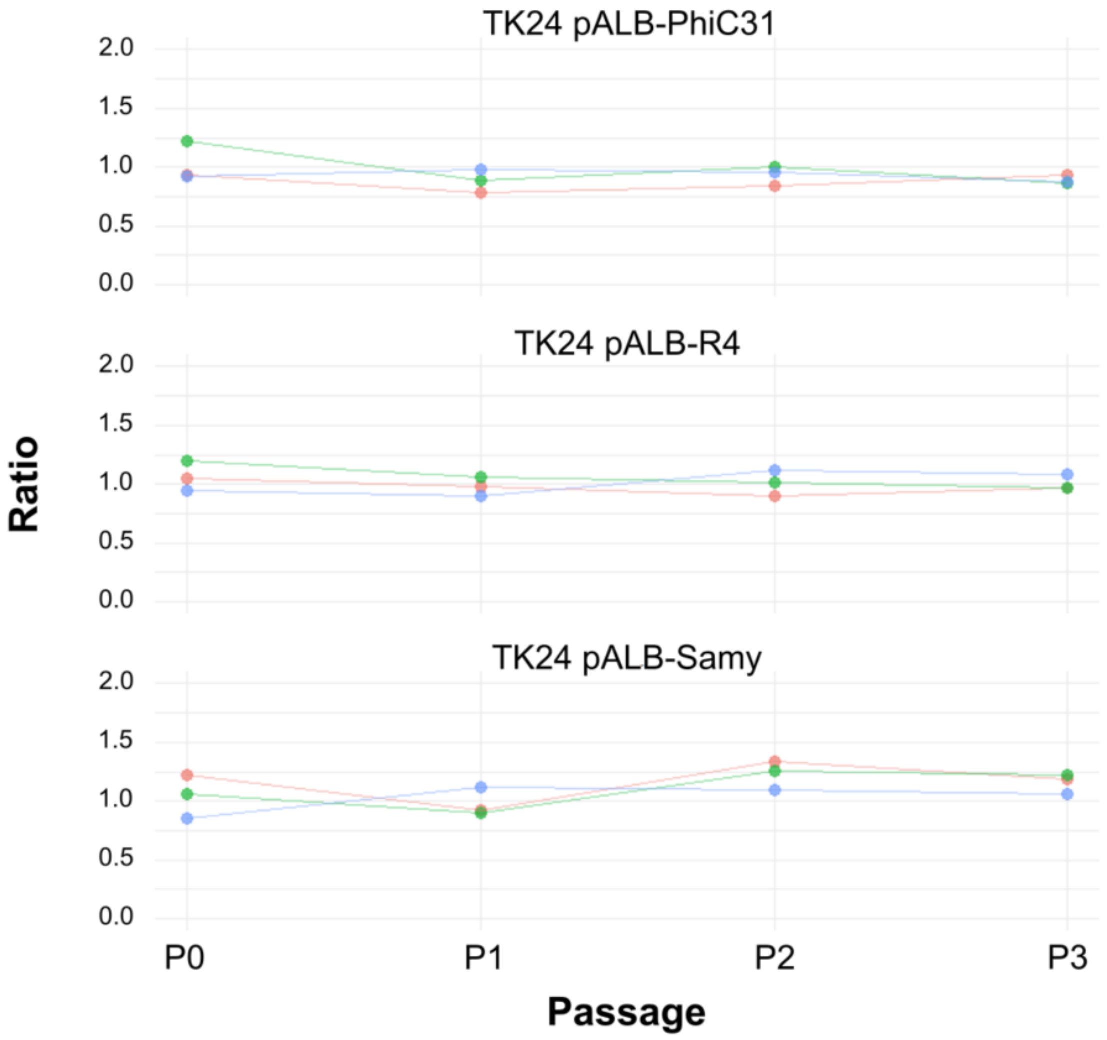

Figure S6: Proportion of strains resistant to apramycin after several passages of *S. lividans* TK24 strains harboring the albonoursin BGC inserted at PhiC31-, R4-, or Samy-*att*B sites

Each point represents an independent clone. For each genetic background, three independent clones underwent three successive weekly passages (P1–P3) on antibiotic-free SFM plates. At each passage, spores were collected, and stocks were titrated by comparing colony-forming units (CFUs) on SFM medium with and without apramycin. The ratio reflects CFUs without antibiotic / CFUs with apramycin. Pairwise comparisons of all passages to the initial time point (P0) using the Wilcoxon rank-sum test (with continuity correction and Benjamini–Hochberg adjusted p-values) revealed no significant differences for any strain.

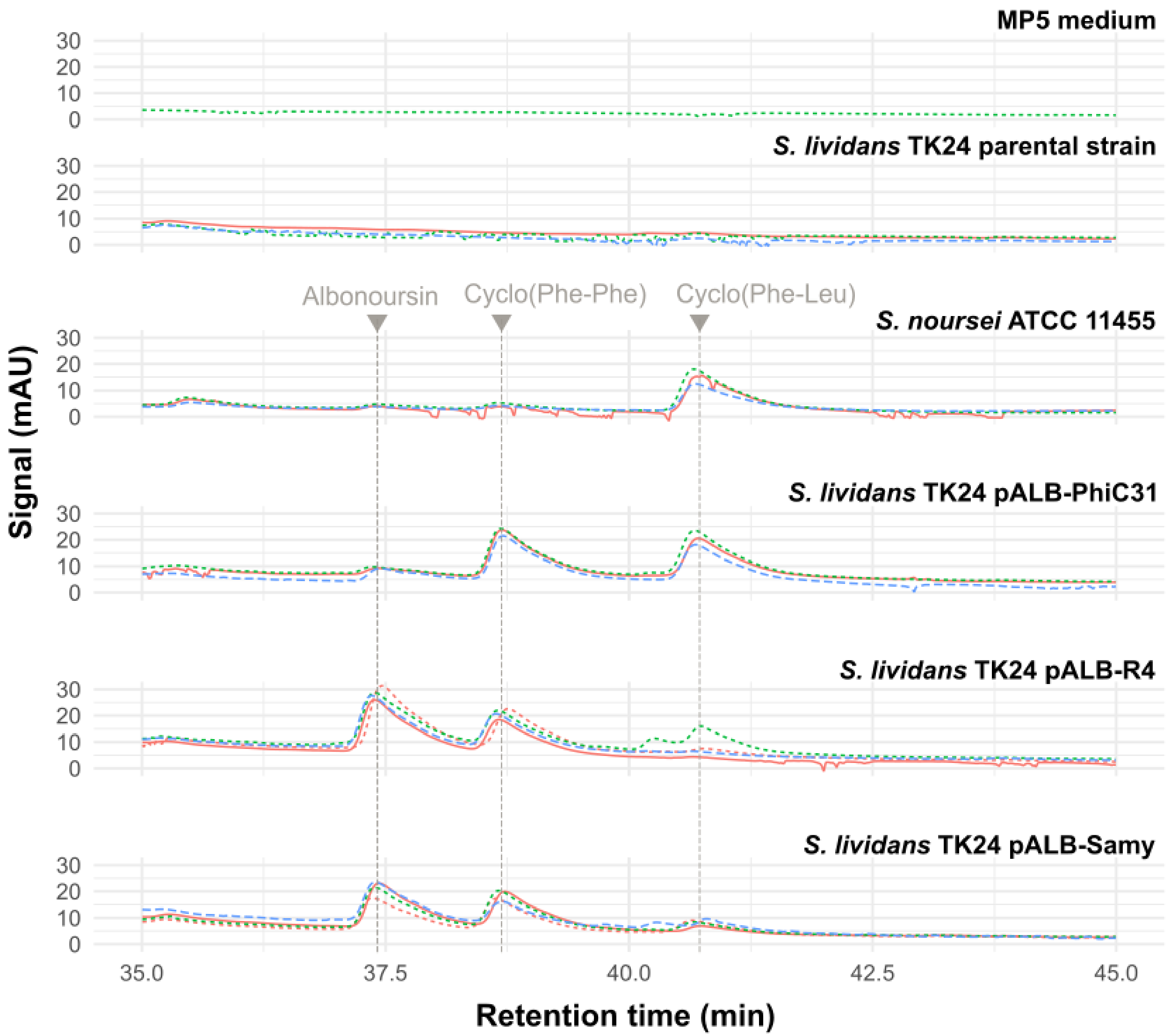

Figure S7: HPLC analysis of specialized metabolites produced by *S. lividans* strains harboring the albonoursin biosynthetic gene cluster at PhiC31-, R4-, or Samy-*att*B sites

Cultures were grown for 4 days in MP5 medium, and the chromatogram illustrates the UV signal intensity (in milli-Absorbance Units, mAU) at 297 nm of detected compounds over retention time. To identify peaks specific to the albonoursin cluster products, supernatants from *Streptomyces noursei* ATCC 11455, the natural producer of albonoursin, and *S. lividans* TK24 parental strain were used as positive and negative controls, respectively, with uninoculated MP5 medium as an additional negative control. The colors represent distinct clones of the genetically engineered *S. lividans* strains harboring the albonoursin cluster at the PhiC31 ("pALB-PhiC31"), R4 ("pALB-R4"), or Samy ("pALB-Samy") insertion sites. The shape of the line indicates independent experiments. The positions of albonoursin cluster products identified by LC-MS analysis (**Fig.S8**) are indicated by vertical lines.

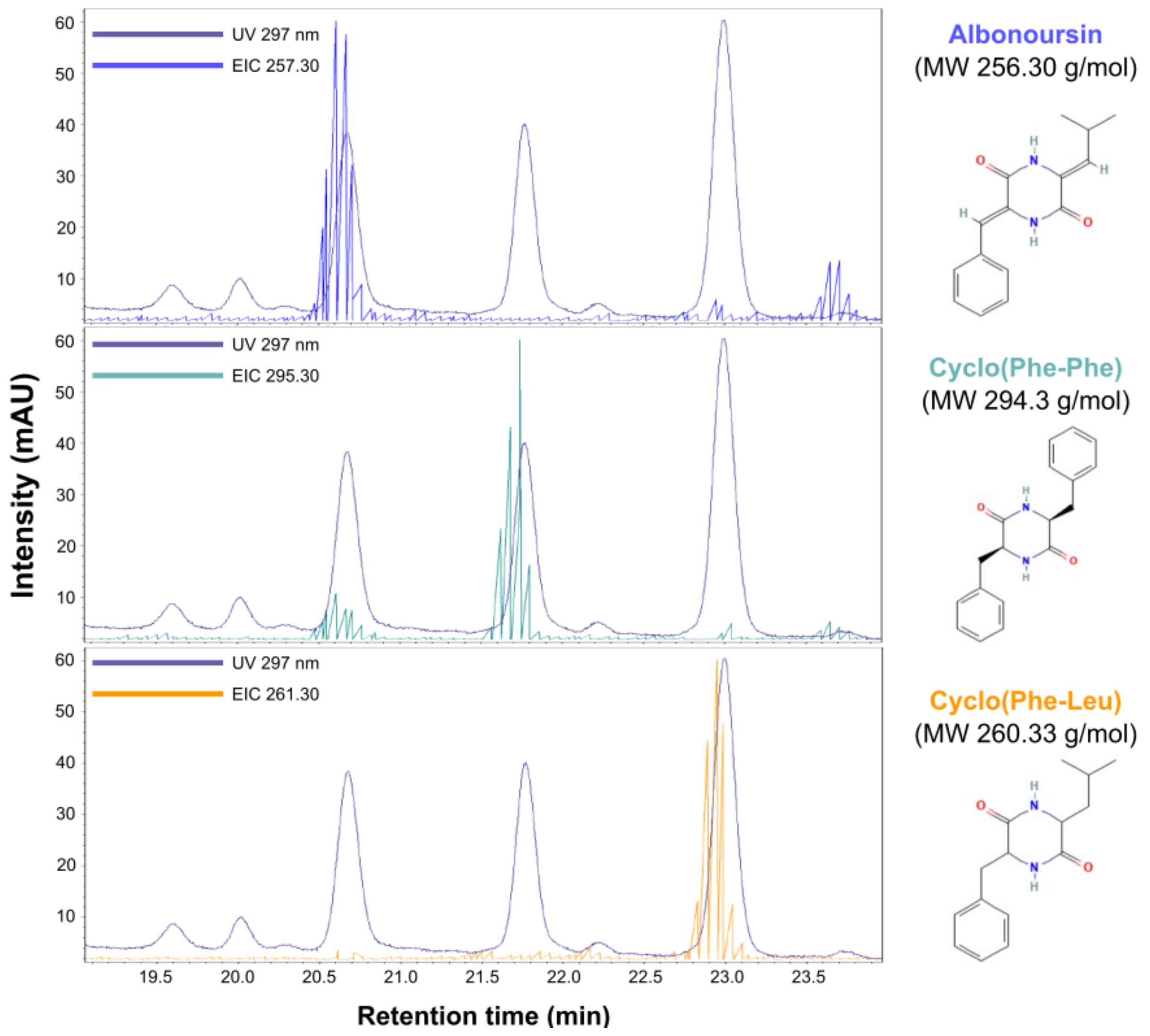

Figure S8: Liquid chromatography-mass spectrometry analysis of the albonoursin and related products

This chromatogram presents the results of a LC-MS analysis, illustrating the intensity (in milli-Absorbance Units, mAU) of detected compounds over time. The dark blue line represents the UV chromatogram at 297 nm, while the colored lines indicate the extracted ion chromatograms (EIC) in positive mode at m/z 257.30 (intense blue), 295.30 (green), and 261.30 (yellow), encompassing all mass spectrometry levels. The analysis was performed using a solid-phase extraction-enriched sample derived from the supernatant of a 4-day culture of the *S. lividans* pALB-Samy strain. The molecular formulas and molecular weights for albonoursin, cyclo(Phe-Phe), and cyclo(Phe-Leu) were sourced from NLM PubChem (https://pubchem.ncbi.nlm.nih.gov/compound/). Please note that the retention times observed in this experiment differ from those in the HPLC analysis presented in **Figure S7**, as the two experiments were conducted under distinct conditions.

Table S1: Strains and plasmids used in this study

Name	Main characteristics	Reference
Streptomyces* strains		
***S. ambofaciens* ATCC 23877**	RP3486 strain deposited at the ATCC by RP; Sequenced genome: GCF_001267885.1_ASM126788v1	(Pinnert-Sindico S, Ninet L, Preud'homme J, Cosar C 1954), genome sequence (Thibessard et al. 2015), Pernodet-Lautru's lab collection
***S. ambofaciens* DSM 40697**	Tü13 strain deposited at the DSM collection; Sequenced genome: CP012949	(Hütter 1967), genome sequence (Thibessard and Leblond 2016), Pernodet-Lautru's lab collection
***S. albidoflavus* J1074**	Sequenced genome: CP004370	(Zaburannyi et al. 2014), Pernodet-Lautru's lab collection
***S. coelicolor* A(3)2**	Sequenced genome: NC_003888.3	(Freeman et al. 1977), Pernodet-Lautru's lab collection
***S. globisporus* NBC_01004**	Sequenced genome: CP109059	Pernodet-Lautru's lab collection
***S. lividans* 66 TK24**	Streptomycin-resistant mutation (*str6)* genetic marker<br>Sequenced genome: CP009124	(Hopwood et al. 1983), Pernodet-Lautru's lab collection
***S. noursei* ATCC 11455**	Albonoursin native producer	Pernodet-Lautru's lab collection
***S. venezuelae* ATCC 10712**	*S. venezuelae* Ehrlich *et al.,* type strain isolated from soil in Venezuela<br>Sequenced genome: CP029197	Pernodet-Lautru's lab collection
***Escherichia coli* strain**		
**DH5α**	General cloning strain	Lab collection
**ET12567 pUZ8002**	Donor strain, whose conjugation helper non transmissible plasmid (pUZ8002) encodes kanamycin resistance, used for the conjugative transfer of DNA from *E. coli* to *Streptomyces*	(Gust et al. 2004)
**Plasmids[§]**		
**pOJ260**	**ColE1, *oriT, lacZ'*, *aac(3)IV***<br>Non-integrative plasmid, replicative in *E. coli* but not *Streptomyces*, carrying the	(Bierman et al. 1992)

	apramycin resistance gene and an origin of transfer for conjugation	
**pOJ260-*SAMYPH94***	**ColE1, *oriT, lacZ'Ω SAMYPH94*, *aac(3)IV*** Samy-based integrative vector, derivative of pOJ260, containing, between the *lac* promoter and *lacZ'* sequence, the 56,510-58,494 genomic region of Samy phage (OR263580.1) including the *SAMYPH94* gene encoding the Samy-integrase, its promoter and Samy-*att*P site sequences	This study
**pOSV802**	**p15A, *oriT, amilCP*, *aac(3)IV, PhiC31 int-attP,* FRT** PhiC31-based integrative vector	(Aubry et al. 2019)
**pOSV819**	**p15A, *oriT, amilCP*, *aac(3)IV, R4 int-attP,* FRT** R4-based integrative vector, new derivative of pOSV vector, containing between *Afl*II and SbfI sites the R4 *att-int* module from pJH1R4 (Gao et al. 2019)	This study
**pOSV876**	**p15A, *oriT, amilCP*, *aac(3)IV, Samy int-attP,* FRT** Samy-based integrative vector, new derivative of pOSV vector, containing between *Afl*II and SbfI sites the 56,510-58,494 genomic region of Samy phage (OR263580.1) including the *SAMYPH94* gene encoding the Samy-integrase, its promoter and Samy-*att*P site sequences	This study
**pCEA007** referred to as **pALB-PhiC31** in this study	**pOSV802::*rpsL*(TP)-*tipA*$_{RBS}$-*albABC*** *rpsL*(TP) promoter and *tipA* RBS driving expression of the albonoursin biosynthetic cluster (*albABC*, 1.8 kb) in place of the *amilCP* expression module	(Aubry et al. 2019)
**pALB-R4**	**pCEA007::*att-int* R4** *att-int* PhiC31 replaced with *att-int* R4 module from pOSV819 into SpeI-SbfI sites	This study
**pALB-Samy**	**pCEA007::*att-int* Samy** *att-int* PhiC31 replaced with *att-int* Samy module from pOSV876 into SpeI-SbfI sites	This study

*Abbreviations: ATCC (American Type Culture Collection), DSM (*Deutsche Sammlung von Mikroorganismen*), RP (Rhône-Poulenc); § *aac(3)-IV*, apramycin resistance gene; *amilCP*, gene coding for an *Acropora millepora* blue chromoprotein; *att*P, phage attachment site; *ColE1*, origin of replication in *E. coli* (not functional in *Streptomyces*); FRT corresponds to the sites recognized by the Flp Recombinase; *int*, phage integrase gene; *lacZ'*, gene encoding the LacZα; *oriT*, origin of transfer; *p15A*, origin of replication in *E. coli* (not functional in *Streptomyces*)

Table S2: Primers used in this study

Primer name	Target region	Sequence (5′-3′)*
**Primers used for vector cloning**		
**SBM375**	pOJ260	TTTGGTCTCCCCTGTGTGAAATTGTTATCC
**SBM376**		CAGCTATGACGGTCTCATGATTACGAATTCGATATCG
**SBM560**	Upstream *SAMYPH94* (56,510-56,529 genomic region of Samy phage OR263580.1 sequence)	GGGGGTCTCTAACACATCGTGAACTTCAGCGTGA
**SBM561**	Downstream *SAMYPH94* (58,477-58,494 genomic region of Samy phage OR263580.1 sequence)	CGCGGTCTCGCAGGGACTCCGGGGCTCTCATG
**SBM588**	Downstream *SAMYPH94* (58,475-58,494 genomic region of Samy phage OR263580.1 sequence)	CCGCTTAAGGACTCCGGGGCTCTCATGAG
**SBM589**	Upstream *SAMYPH94* (56,510-56,528 genomic region of Samy phage OR263580.1 sequence)	TTCCCTGCAGGCATCGTGAACTTCAGCGTG
**HL-R4-SbfI**	R4 *att-int* module from pJH1R4 (Gao et al. 2019)	TCCGTCGACCTGCAGGCATGCAAG
**HL-R4-AflII**		ACTTAAGCTACTCGGCCACGTCTCGCCAC
**Primers used for Samy-*att*L and Samy-*att*R region amplifications**		
**SBM584**	Samy-*att*P region on pOJ260-*SAMYPH94*, divergent primer used to amplify *att*R	CTGCAGGTCGACTCTAGAGG
**SBM585**	Samy-*att*P region on pOJ260-*SAMYPH94*, divergent primer used to amplify *att*L	CCTCCGCTTGAATGACGAAG
**HL15**	*S. ambofaciens* DSM 40697 and ATCC 23877 strain genomic regions located upstream Samy-*att*B site, used to amplify Samy-*att*L	GATCAGCGTGCCCTTGGT
**HL16**	*S. ambofaciens* DSM 40697 and ATCC 23877 strain genomic regions located downstream Samy-*att*B site, used to amplify Samy-*att*R	TCCTCCAGGGCCTGGTTC
**SBM582**	*S. coelicolor* A(3)2 and *S. lividans* TK 24 strain genomic regions used to amplify Samy-*att*R	CCTCAATGCGCTGGGAGAA
**SBM583**	*S. coelicolor* A(3)2 and *S. lividans* TK 24 strain genomic regions used to amplify Samy-*att*L	CACCCGGTTCAGGATCGAG

**SBM590**	*S. albidoflavus* J1074 genomic region used to amplify Samy-*att*R	ATGTCCACTTCCTCCAGCAC
**SBM591**	*S. albidoflavus* J1074 genomic region used to amplify Samy-*att*L	GGTAGCAGTTGACGTTGGTG
**SBM592**	*S. globisporus* NBC_01004 genomic region used to amplify Samy-*att*R	ATGTGGAGCCGGTTCTTGAG
**SBM593**	*S. globisporus* NBC_01004 genomic region located used to amplify Samy-*att*L	CTTGACGACGATCTGGGAGT
**SBM594**	*S. venezuelae* ATCC 10712 genomic region used to amplify Samy-*att*R	ATTGACTTCCTCCAGCTCGT
**SBM595**	*S. venezuelae* ATCC 10712 genomic region used to amplify Samy-*att*L	TCTTGACGACGATCTGGGAG
**Primers used to check the proper insertion of pALB plasmids in *S. lividans* TK 24**		
**SBM688**	Sequence located within pALB plasmids, used with SBM582, SBM 686 or SBM690 to check the insertion at Samy-, PhiC31- and R4-*att*B sites, respectively	GTAACAGATGAGGGCAAGCG
**SBM686**	*S. lividans* TK 24 strain genomic regions used to amplify PhiC31-*att*R	CCAGGTGGCAAGAAGTTCAA
**SBM690**	*S. lividans* TK 24 strain genomic regions used to amplify R4-*att*R	GAGTACGTCCTCCAGCAGTC
**Primers used for qPCR analyses**		
**SBM609**	Samy-*att*P junction	CGGGGCTTCTTTACGTCCTT
**SBM615**	Samy-*att*P junction	GTAGAGGCTCGCACGCTT
**SBM611**	*SAMYPH94* (Samy integrase)	GACGTCCTCGTCAACCCTG
**SBM612**	*SAMYPH94* (Samy integrase)	ACCTCCGCTTGAATGACGAA

* Restriction sites are underlined.

Table S3: Individual *att*B sites for site-specific recombination systems developed to genetically engineered *Streptomyces* chromosome

<table>
<tr><th>Name</th><th>attB sequence
(5′-3′)</th><th>Source of the
attB sequence</th><th>Ref.</th></tr>
<tr><td>µ1/6</td><td>TCTTGTAAAGCGGGGGTCGTCGGTTCGAA
CCCGACAGGGGGCTCAG</td><td>Streptomyces aureofaciens B96</td><td>(Farkašovská and Godány 2012)</td></tr>
<tr><td>PhiBT1</td><td>CCAGGTTTTTGACGAAAGTGATCCAGATG
ATCCAGC</td><td>Streptomyces coelicolor M145</td><td>(Gregory et al. 2003)</td></tr>
<tr><td>PhiC31</td><td>GGTGCCAGGGCGTGCCCTTGGGCTCCCCG
GGCGCG</td><td>Streptomyces lividans</td><td>(Groth et al. 2000)</td></tr>
<tr><td>PhiJoe</td><td rowspan="3">ATCTGGATGTGGGTGTCCATCTGCGGGCA
GACGCCGCAGTCGAAGCACGG</td><td rowspan="3">Streptomyces venezuelae ATCC 10712</td><td>(Fogg et al. 2017)</td></tr>
<tr><td>PhiOZJ</td><td rowspan="2">(Ko et al. 2020)</td></tr>
<tr><td>PhiWTR</td></tr>
<tr><td>pSAM2</td><td>GCGCGCTTCGTTCGGGACGAAGAGGT</td><td>Streptomyces ambofaciens DSM 40697</td><td>(Boccard et al. 1989; Raynal et al. 1998)</td></tr>
<tr><td>R4</td><td>AGTTGCCCATGACCATGCCGAAGCAGTGG
TAGAAGGGCACCGGCAGACAC</td><td>Streptomyces parvulus 2297</td><td>(Miura et al. 2011)</td></tr>
<tr><td>Samy</td><td>GAAGGTGATGCCGCGGAACTCGGGTGTG
TCGACGGTGCGGGTGGTGACCG</td><td>Streptomyces ambofaciens DSM 40697</td><td>This study</td></tr>
<tr><td>SV1</td><td>CATCAGGGCGGTCAGGCCGTAGATGTGG
AAGAACGGCAGCACGGCGAGGACG</td><td>Streptomyces coelicolor A3(2)</td><td>(Fayed et al. 2014)</td></tr>
<tr><td>TG1</td><td>GATCAGCTCCGCGGGCAAGACCTTCTCCT
TCACGGGGTGGAAGGTC</td><td>Streptomyces avermitilis ATCC 31267</td><td>(Morita et al. 2009)</td></tr>
<tr><td>VWB</td><td>CTCTCCTAAAGCGGGTGTCGCAGGTTCGA
ATCCTGCCGGGGGCAC</td><td>Streptomyces venezuelae ETH14630</td><td>(Van Mellaert et al. 1998)</td></tr>
</table>

References

Aubry C, Pernodet JL, Lautru S (2019) Modular and Integrative Vectors for Synthetic Biology Applications in *Streptomyces* spp. Appl Environ Microbiol 85. https://doi.org/10.1128/AEM.00485-19

Bierman M, Logan R, O'Brien K, Seno ET, Rao RN, Schoner BE (1992) Plasmid cloning vectors for the conjugal transfer of DNA from *Escherichia coli* to *Streptomyces* spp. Gene 116:43–9

Boccard F, Smokvina T, Pernodet JL, Friedmann A, Guérineau M (1989) The integrated conjugative plasmid pSAM2 of *Streptomyces ambofaciens* is related to temperate bacteriophages. The EMBO Journal 8:973–980. https://doi.org/10.1002/j.1460-2075.1989.tb03460.x

Di Salvo M (2017) MarcoDiSalvo90/G4PromFinder: Third release of G4PromFinder software

Di Salvo M, Pinatel E, Talà A, Fondi M, Peano C, Alifano P (2018) G4PromFinder: an algorithm for predicting transcription promoters in GC-rich bacterial genomes based on AT-rich elements and G-quadruplex motifs. BMC Bioinformatics 19:36. https://doi.org/10.1186/s12859-018-2049-x

Farkašovská J, Godány A (2012) Analysis of the Site-Specific Integration System of the *Streptomyces aureofaciens* Phage μ1/6. Curr Microbiol 64:226–233. https://doi.org/10.1007/s00284-011-0054-7

Fayed B, Younger E, Taylor G, Smith MCM (2014) A novel Streptomyces spp. integration vector derived from the *S. venezuelae* phage, SV1. BMC Biotechnol 14:51. https://doi.org/10.1186/1472-6750-14-51

Fogg PCM, Haley JA, Stark WM, Smith MCM (2017) Genome Integration and Excision by a New *Streptomyces* Bacteriophage, ϕJoe. Appl Environ Microbiol 83:e02767-16. https://doi.org/10.1128/AEM.02767-16

Freeman RF, Bibb MJ, Hopwood DA (1977) Chloramphenicol Acetyltransferase-independent Chloramphenicol Resistance in *Streptomyces coelicolor* A3(2). Journal of General Microbiology 98:453–465. https://doi.org/10.1099/00221287-98-2-453

Gao H, Murugesan B, Hoßbach J, Evans SK, Stark WM, Smith MCM (2019) Integrating vectors for genetic studies in the rare Actinomycete *Amycolatopsis marina*. BMC Biotechnology 19:32. https://doi.org/10.1186/s12896-019-0521-y

Gregory MA, Till R, Smith MCM (2003) Integration Site for *Streptomyces* Phage φBT1 and Development of Site-Specific Integrating Vectors. J Bacteriol 185:5320–5323. https://doi.org/10.1128/JB.185.17.5320-5323.2003

Groth AC, Olivares EC, Thyagarajan B, Calos MP (2000) A phage integrase directs efficient site-specific integration in human cells. Proc Natl Acad Sci USA 97:5995–6000. https://doi.org/10.1073/pnas.090527097

Gust B, Chandra G, Jakimowicz D, Yuqing T, Bruton CJ, Chater KF (2004) Lambda red-mediated genetic manipulation of antibiotic-producing *Streptomyces*. Adv Appl Microbiol 54:107–28. https://doi.org/10.1016/S0065-2164(04)54004-2

Hopwood DA, Kieser T, Wright HM, Bibb MJ (1983) Plasmids, Recombination and Chromosome Mapping in *Streptomyces lividans* 66. Microbiology 129:2257–2269. https://doi.org/10.1099/00221287-129-7-2257

Hütter R (1967) [Classification of the streptomycetes with special regard to the antibiotics formed from them]. Bibl Microbiol 6:1–382

Ko B, D'Alessandro J, Douangkeomany L, Stumpf S, deButts A, Blodgett J (2020) Construction of a new integrating vector from actinophage ϕOZJ and its use in multiplex *Streptomyces* transformation. Journal of Industrial Microbiology and Biotechnology 47:73–81. https://doi.org/10.1007/s10295-019-02246-7

Lorenzi J-N, Thibessard A, Lioy VS, Boccard F, Leblond P, Pernodet J-L, Bury-Moné S (2022) Ribosomal RNA operons define a central functional compartment in the *Streptomyces* chromosome. Nucleic Acids Research 50:11654–11669. https://doi.org/10.1093/nar/gkac1076

Miura T, Hosaka Y, Yan-Zhuo Y, Nishizawa T, Asayama M, Takahashi H, Shirai M (2011) I*n vivo* and *in vitro* characterization of site-specific recombination of actinophage R4 integrase. J Gen Appl Microbiol 57:45–57. https://doi.org/10.2323/jgam.57.45

Morita K, Yamamoto T, Fusada N, Komatsu M, Ikeda H, Hirano N, Takahashi H (2009) The site-specific recombination system of actinophage TG1. FEMS Microbiology Letters 297:234–240. https://doi.org/10.1111/j.1574-6968.2009.01683.x

Pinnert-Sindico S, Ninet L, Preud'homme J, Cosar C (1954) A new antibiotic Spiramycin. Antibiotics annual p 724-727

Rangannan V, Bansal M (2009) Relative stability of DNA as a generic criterion for promoter prediction: whole genome annotation of microbial genomes with

varying nucleotide base composition. Mol BioSyst 5:1758. https://doi.org/10.1039/b906535k

Raynal A, Tuphile K, Gerbaud C, Luther T, Guérineau M, Pernodet J (1998) Structure of the chromosomal insertion site for pSAM2: functional analysis in *Escherichia coli*. Molecular Microbiology 28:333–342. https://doi.org/10.1046/j.1365-2958.1998.00799.x

Thibessard A, Haas D, Gerbaud C, Aigle B, Lautru S, Pernodet JL, Leblond P (2015) Complete genome sequence of *Streptomyces ambofaciens* ATCC 23877, the spiramycin producer. J Biotechnol 214:117–8. https://doi.org/10.1016/j.jbiotec.2015.09.020

Thibessard A, Leblond P (2016) Complete Genome Sequence of *Streptomyces ambofaciens* DSM 40697, a Paradigm for Genome Plasticity Studies. Genome Announc 4:e00470-16. https://doi.org/10.1128/genomeA.00470-16

Van Mellaert L, Mei L, Lammertyn E, Schacht S, Ann J (1998) Site-specific integration of bacteriophage VWB genome into *Streptomyces venezuelae* and construction of a VWB-based integrative vector. Microbiology 144:3351–3358. https://doi.org/10.1099/00221287-144-12-3351

Zaburannyi N, Rabyk M, Ostash B, Fedorenko V, Luzhetskyy A (2014) Insights into naturally minimised *Streptomyces albus* J1074 genome. BMC Genomics 15:97. https://doi.org/10.1186/1471-2164-15-97
